# Efficacy of cognitive behavioral therapy in treating repetitive negative thinking, rumination, and worry – a transdiagnostic meta-analysis

**DOI:** 10.1017/S0033291725000017

**Published:** 2025-02-07

**Authors:** Kilian Leander Stenzel, Joshua Keller, Lukas Kirchner, Winfried Rief, Max Berg

**Affiliations:** Department of Psychology, Division of Clinical Psychology & Psychotherapy, Philipps-University of Marburg, Marburg, Germany

**Keywords:** cognitive behavioral therapy, meta-analysis, repetitive negative thinking, rumination, transdiagnostic, worry

## Abstract

Repetitive negative thinking (RNT) is a transdiagnostic process associated with the onset, maintenance, and risk of relapse of various mental disorders. However, previous research syntheses addressing the effect of cognitive behavioral therapy (CBT) on RNT are limited to specific diagnoses, treatments, or RNT constructs (transdiagnostic RNT, worry, rumination). In the present meta-analysis, we integrate findings from randomized controlled trials (RCTs) of CBT on RNT across diagnoses, intervention types, and RNT constructs. We investigate the following questions: What is the overall transdiagnostic efficacy of CBT interventions on all post-treatment RNT outcomes? Which RNT construct is addressed most effectively? Are RNT-specific treatments superior in reducing RNT than less specific approaches? Inclusion criteria were met by 55 studies with a total of 4,970 participants. The overall post-treatment effect of CBT interventions on RNT compared to respective control groups was moderate in favor of CBT (*g* = −0.67). Treatment efficacy did not differ significantly by RNT construct. RNT-specific interventions (*g* = −0.99) were significantly more efficacious in reducing RNT than less specific approaches (*g* = −0.56). Treatment efficacy was not significantly enhanced by individual or in-person settings. Our results advocate a dissemination of RNT-specific treatments in research and practice and a general improvement of CBT treatments by focusing on relevant transdiagnostic processes such as RNT.

## Introduction

Mental disorders such as depressive or anxiety disorders are still highly prevalent across cultures (GBD 2019 Mental Disorders Collaborators, 2022; Kessler et al., 2009). In 2019, 16% of disability-adjusted life years (DALYs) were attributed to mental disorders causing an estimated economic burden of about 5 trillion U.S. Dollar per year (Arias, Saxena, & Verguet, 2022). Hence, the improvement of existing psychological treatments is exceedingly relevant.

### Classification of psychopathology

For decades, clinical psychology and psychiatry have conceptualized mental disorders with a “latent disease model” assuming underlying psychopathological entities (Hofmann, 2014; Rief et al., 2023) having generated many diagnostic categories manifested in standardized inventories such as the Diagnostic and Statistical Manual of Mental Disorders (DSM-5; American Psychiatric Association, 2013) or the International Statistical Classification of Diseases and Related Health Problems, Section on Mental, Behavioral and Neurodevelopmental Disorders (ICD-11; WHO, 2022). Based on this model, researchers developed disorder-specific models and corresponding treatments aiming at relieving “disorder-specific” symptoms (Ehring & Behar, 2020; Harvey, Watkins, Mansell, & Shafran, 2004). However, many treatments still neglect the substantial rates of comorbidity in most patient populations (Borsboom, 2002; Kessler et al., 2011), which is why a focus on common processes of psychopathology has been endorsed, as research nowadays advocates that most mental disorders share underlying “transdiagnostic” processes (Harvey et al., 2004; Mansell, Harvey, Watkins, & Shafran, 2008; Sauer-Zavala et al., 2017). Consequently, it has been recommended to also extend meta-research to transdiagnostic processes (Rief et al., 2024).

### Repetitive negative thinking as a transdiagnostic process and mediator of symptom improvement

Repetitive negative thinking (RNT) poses such a transdiagnostic process being defined as a thinking process that is repetitive, passive, and/or relatively uncontrollable, and focused on negative content (Ehring & Watkins, 2008). RNT is an established risk factor for the onset and maintenance of various mental disorders (Ehring & Watkins, 2008; Taylor & Snyder, 2021; Watkins & Roberts, 2020) and increases with the degree of comorbidity in patients with comorbid major depressive disorder (MDD) and anxiety disorders (McEvoy, Watson, Watkins, & Nathan, 2013). It also mediated disorder shifts of anxiety to MDD and *vice versa* in a prospective study (Spinhoven, Van Hemert, & Penninx, 2018). Furthermore, RNT predicted unique variance in MDD and anxiety symptom improvement (Kertz, Koran, Stevens, & Björgvinsson, 2015), decreased therapy success (Kertz et al., 2015), and a higher risk of relapse after therapy for MDD (Michalak, Hölz, & Teismann, 2011). Studies suggest that the thought *process* itself is more important than the thought *content* (Bell, Marx, et al., 2023; Rosenkranz, Takano, Watkins, & Ehring, 2020) in predicting psychopathology (McEvoy et al., 2018; Spinhoven, Klein, et al., 2018; Topper, Molenaar, Emmelkamp, & Ehring, 2014). Nevertheless, the definition of RNT also entails content-oriented constructs such as rumination — thoughts and behaviors that “repetitively focus an individual’s attention on his or her negative feelings, and the nature and implications of these feelings (including the causes, meanings, and consequences of the feelings)” (Harvey et al., 2004, p. 196; Nolen-Hoeksema, 1991), and worry — a future-oriented mental problem-solving attempt generating a “chain of thoughts and images, negatively affect-laden and relatively uncontrollable” (Borkovec, Ray, & Stober, 1998; Borkovec, Robinson, Pruzinsky, & DePree, 1983). Historically, rumination and worry were investigated and treated as two separate constructs. However, multiple cross-sectional and factor-analytical studies found a common RNT factor underlying rumination and worry (McEvoy et al., 2010; Taylor & Snyder, 2021; Topper et al., 2014), so that they are considered largely overlapping constructs today. Considering theoretical and empirical accounts of rumination and worry, it seems reasonable to subsume both constructs under the process-focused definition of transdiagnostic RNT.

### How RNT is addressed by CBT

CBT is the most studied psychological therapy (Cuijpers, Harrer, Miguel, Ciharova, & Karyotaki, 2023) being widely considered a highly effective treatment for many mental disorders (David, Cristea, & Hofmann, 2018). CBT is a class of interventions sharing the rationale that maladaptive cognitions and behavioral strategies contribute to the maintenance of mental disorders (Beck, 1970). Today, CBT comprises core components such as cognitive restructuring and components targeting specific symptoms of mental disorders such as behavioral activation for depression or exposure to anxiety disorders (Cuijpers, Miguel, et al., 2023). These core components are mostly not restricted to single diagnoses but pose fundamentally transdiagnostic treatment ideas (e.g., functional analysis, behavioral experiments). The cognitive-behavioral framework also yielded so-called “third wave” therapies taking a transdiagnostic approach by focusing on the management of thoughts and emotions through observation, acceptance, cognitive defusion, and mindfulness practice (Hayes, Strosahl, & Wilson, 2003). Another transdiagnostic therapy addressing thought processes is Metacognitive Therapy (MCT, Wells et al., 2009) focusing on metacognitions about uncontrollability, perceived threat, and the importance of thinking and feeling. It seeks to change metacognitions about the usefulness of cognitive engagement using mindfulness and behavioral interventions. However, these treatments were not explicitly designed to address RNT according to the current frameworks (Ehring & Watkins, 2008). Recently, effective treatments were derived from the RNT framework and explicitly target relevant processes. Such treatments include rumination-focused CBT (Watkins, 2018) or worry-specific internet-based interventions (Dahlin, Johansson, Romare, Carlbring, & Andersson, 2022). Unfortunately, these developments are only partially reflected in the scientific literature on the impact of CBT on RNT, as there remain gaps in comparative research exploring the extent to which CBT interventions reduce RNT and whether differences exist.

### Previous meta-analyses on the effect of psychological treatments on RNT

Although RNT is an established transdiagnostic process encompassing rumination and worry, previous meta-analyses investigating RNT rarely investigated it as such. Instead, they selectively focused on single mental disorders such as anxiety disorders (Hall, Kellett, Berrios, Bains, & Scott, 2016; Monteregge, Tsagkalidou, Cuijpers, & Spinhoven, 2020; Olatunji, Davis, Powers, & Smits, 2013) or depression (Spinhoven, Klein, et al., 2018), single intervention types (e.g., mindfulness; Li et al., 2022; Mao, Li, Wu, Luo, & Hu, 2023; Perestelo-Perez, Barraca, Peñate, Rivero-Santana, & Alvarez-Perez, 2017), or single RNT subconstructs such as worry (Covin, Ouimet, Seeds, & Dozois, 2008; Hanrahan, Field, Jones, & Davey, 2013). Herby, Spinhoven, Klein, et al. (2018) could show that CBT for depression decreased RNT significantly and moderately. Interestingly, RNT-focused treatments (*g* = 0.53) did not differ significantly from traditional CBT (*g* = 0.63), while being descriptively even less effective. The authors included rf-CBT, Cognitive Control Training, Concreteness Training, and Mindfulness-based Cognitive Therapy (MBCT) as RNT-specific treatments. Another interesting finding was that the effect of any treatment on RNT was not significantly affected by the type of RNT measured (rumination only: *g* = 0.49, rumination-only ruminative response scale: *g* = 0.54, worry: *g* = 0.42, content-independent RNT: *g* = 0.77). Extending these findings, Monteregge et al. (2020) showed that RNT-focused (*g* = 0.69) and non-RNT-focused (*g* = 0.58) psychological anxiety interventions also did not differ significantly with respect to RNT (including rumination, worry, and content-independent RNT). Here, RNT-focused interventions were defined broadly including MCT, mindfulness-based therapies, emotion regulation therapy, worry extinction therapy, worry journaling, positive worry alternatives, intolerance of uncertainty training, working memory training, attentional bias training, behavioral activation for worry, or a combination thereof. Furthermore, Bell, Marx, et al. (2023) also found that RNT-focused (*g* = 0.50) and non-RNT-focused (*g* = 0.52) treatments did not differ significantly (*p* = .06) with respect to their effect on RNT in a juvenile sample. However, treatments seeking to change the process of RNT (*g* = 0.85) had significantly greater effects on RNT than those seeking to change negative thought content itself (*g* = 0.13). In this study, RNT-focused interventions were defined by whether “the intervention explicitly targeted either the process of repeated attention towards negative thoughts (e.g. mindfulness or acceptance approaches) or the content of negative biases that perpetuated repeated focus on negative thoughts” (Bell, Marx, et al., 2023, p. 3).

Taken together, previous meta-analyses have typically focused on specific mental disorders, intervention types, or outcomes. Our meta-analysis aims to draw a broader picture by including various mental disorders, CBT interventions, and RNT conceptualizations, thus adopting a more transdiagnostic approach to RNT. This approach allows us to examine the generalizability of previous findings and to investigate both cross-disorder commonalities and disorder-specific differences. Furthermore, previous studies specified the RNT focus of interventions based on whether the overarching treatment rationale addressed RNT – a well-established approach that acknowledges the broad theoretical underpinnings of respective treatments. However, in practice, treatments derived from these rationales can vary considerably depending on how they are adapted for and monitored within individual studies. Oftentimes, this variability cannot even be quantified, because treatment fidelity is rarely assessed (Perepletchikova, Treat, & Kazdin, 2007). As a consequence, study authors might not address the constructs the protocol inventors had in mind when establishing the protocol. Therefore, following the suggestion of Rief et al. (2024, pp. 9–10), we sought to acknowledge the study authors’ interpretation of their interventions’ purposes by screening original studies for explicit statements of RNT focus. This offers the opportunity to focus on treatments and interventions with a direct, clearly described, and intended RNT focus.

In sum, our meta-analysis sought to investigate the overall transdiagnostic efficacy of CBT treatments on a broad range of RNT outcomes, such as worry, rumination, and content-independent RNT. Further, it sought to elucidate which RNT subconstruct is addressed most efficaciously and to investigate the difference between RNT-specific and general treatments. We also aimed to investigate potential moderators, such as therapy setting, type of control group, therapy duration, and publication year.

## Methods

The review followed the PRISMA guidelines (Page et al., 2021) and was prospectively registered at PROSPERO (CRD42023453038) and OSF (DOI: 10.17605/OSF.IO/WDBFJ).

### Identification and selection of studies

The following databases were examined in September 2023: EMBASE, PsycINFO via EBSCO, CENTRAL, Web of Science, and PubMed/Medline. We used preregistered search terms including an exhaustive list of mental disorders, CBT treatments, and RNT-related terms (see SI1 in the Supplementary Material). We also screened the reference lists of 10 previous reviews to expand our study pool (SI2 in the Supplementary Material). Studies were eligible if they met all of the following criteria: (a) Included human participants aged 18 years or older with (b) a current mental disorder according to a standardized diagnostic inventory (DSM-IV, DSM-5, ICD-10, ICD-11, and respective text revisions) who (c) received some form of CBT (treatment group), (d) included a control group, (e) used randomized group assignment, (f) were written in English or German, and (g) were published in peer-reviewed journals. CBT treatment was defined as any intervention that explicitly addresses either cognitions, meta-cognitions, and/or behavior in a systematic and standardized manner. It also had to be explicitly designed to build a cognitive or behavioral skill in participants. Thus, behavioral therapies, cognitive and meta-cognitive approaches, emotion regulation-focused interventions, and mindfulness-based treatments were all included in this study (see next section for the final treatments included). In line with our focus on transdiagnostic processes of psychopathology, the outcome variable of interest was RNT and its subconstructs rumination and worry. We only included studies using established questionnaires with at least moderate psychometric properties, and convergent, and predictive validity. A full list of all outcome measures can be found in the SI3 in the Supplementary Material.

After duplicate removal, two study authors K.L.S. *and* M.B. *or* J.K. (note that italic “and” and “or” indicates logical operators with “and” considered before “or”) screened titles and abstracts for inclusion/exclusion criteria in two separate steps. Afterward, both authors reached an agreement on eligible studies by discussion. The remaining full texts were reviewed in detail for compliance with the eligibility criteria. Again, both authors discussed which articles they could agree on.

### Data extraction and variable coding

Two authors (K.L.S. *and* M.B. *or* J.K.) extracted data for each eligible study independently. In case of missing data, corresponding and/or senior authors were contacted twice. After data extraction was completed, mistakes were ruled out, and coding was unified. The spreadsheet containing all extracted data is available as supplementary data on OSF. We extracted the following variables for meta-regressions and subgroup analyses: (1) Treatment specificity: Treatment arms were coded as “specific,” if the study explicitly stated that at least one intervention addressed RNT, worry, or rumination referring to the relevant concept. Otherwise, it was coded as “general.” Hence, RNT-specific treatments included: ACT, CBT, CBT-intolerance of uncertainty, worry exposure, competitive memory training, internet-based CBT, intolerance of uncertainty training, and worry protocols, whereas “general” treatments included: Acceptance-Based Behavior Therapy, anxiety programs, Applied Relaxation, CBT, CBT - imagery rescripting, CBT - verbal restructuring, computerized CBT, Emotion Regulation Therapy, MBCT, mindfulness-based interventions, Mindfulness-Based Stress Reduction, MCT, Unified Protocol; for an overview of these variables, refer to SI7 in the Supplementary Material; (2) setting A: Individual versus group therapy settings; setting B: In-person versus other (e.g., online) settings; (3) type of control group: The comparator type was coded as “passive” in case of a wait-list control, attention control without additional interventions, and treatment-as-usual (TAU) without actively offered intervention or if TAU was unspecified. Control groups that received at least attention, placebo, active medication, neurostimulation, psychoeducation, or non-CBT therapy were coded as “active”; (4) therapy length (number of sessions); (5) publication year. Since no study reported homogenous inpatient samples, we neglected this variable, albeit it was preregistered. For a comprehensive overview of study classifications, variables, and data, refer to SI6 in the Supplementary Material.

### Handling multiple outcomes, treatments, controls, and outliers

Twenty studies in our dataset reported more than one RNT effect size. Six studies used two measures of RNT. In this case, we retained the effect sizes for RNT, rumination, and worry in decreasing priority, whereby we also mitigated frequency imbalances. Furthermore, 10 studies in our dataset reported multiple active treatment groups and four other studies used multiple control groups. Hence, the effect size data were not independent. However, we report a sensitivity analysis of the most conservative and most liberal effect size estimates for multiple control and treatment groups, respectively (see Table 1). Yet, descriptive statistics refer to the sample that only contains independent (unique) samples by not including the same control (in case of multiple treatments) or the same treatment group twice (in case of multiple controls). Studies were detected as influential cases (outliers) if their removal in a leave-one-out sensitivity analysis yielded a Cook’s Distance > 0.45 *or* a DFFITS*_k_*-value > 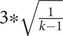 *or* a hat*_k_*-value > 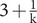 according to Viechtbauer (2010). As we did not anticipate these circumstances, the procedures for handling multiple outcomes and outliers were not preregistered.

**Table 1.** Effects of cognitive behavioral therapy on repetitive negative thinking compared with control groups at post-treatment

Variable	Levels	*k^a^*	*g^b^*	95% CI	*I^2^* (%)	*p^c^*
Overall effect		67	−0.67	[−0.82; −0.53]	80	
Sensitivity analyses						
Multiple treatment*s*	Smaller effect – conservative	57	−0.65	[−0.81; −0.48]	81	
	Larger effect – liberal	57	−0.70	[−0.87; −0.53]	82	
Multiple control*s*	Smaller Effect – conservative	63	−0.70	[−0.85; −0.55]	79	
	Larger effect – liberal	63	−0.72	[−0.86; −0.58]	74	
Risk of Bias	High	26	−0.68	[−0.89; −0.47]	58	.961
	Medium	30	−0.65	[−0.90; −0.40]	87	
	Low	11	−0.65	[−0.89; −0.39]	57	
Outcome	RNT	6	−0.60	[−0.98; −0.22]	59	.522
	Rumination	14	−0.55	[−0.76; −0.34]	50	
	Worry	47	−0.70	[−0.89; −0.52]	84	
Subgroup analyses						
Treatment specificity	General	50	−0.56	[−0.73; −0.39]	80	.002*
	Specialized	17	−0.99	[−1.21; −0.76]	55	
Setting A	Group	28	−0.53	[−0.77; −0.28]	84	.094
	Individual	39	−0.77	[−0.95; −0.60]	73	
Setting B	In-person	50	−0.72	[−0.91; −0.52]	83	.243
	Other^d^	17	−0.57	[−0.74; −0.0]	60	
Type of control group	Active	27	−0.46	[−0.73; −0.20]	84	.021*
	Passive	40	−0.81	[−0.96; −0.65]	64	

*Note*: ^a^*k* indicates number of comparisons for each level. ^b^*g* indicates Hedge’s *g.* ^c^*p* indicates whether the effect sizes of subgroups differed significantly from each other based on a *Q*-test. ^d^Other indicates internet-based therapy with or without in-person support, phonecall, videocall, or mixed therapy (in-person and phonecall).

* *p <* .05.

### Quality assessment

Studies’ risk of bias was determined using the Cochrane Collaboration risk of bias tool (Sterne et al., 2019) with the following criteria: (A) generation of the allocation sequence (proper versus dubious), (B) concealment of the allocation sequence (concealed versus visible), (C) blinding of participants, study personnel (raters, clinicians, statisticians), (D) incomplete outcome data (total ratio and imbalances), (E) selective outcome reporting (differing methods/results or paper/preregistration). Studies with issues on the generation and/or concealment of the allocation sequence were labeled “high risk of bias.” Studies with proper randomization and concealment, but with issues with blinding, completeness of data, and/or outcome reporting were labeled “medium risk of bias.” Studies with no such issues were labeled “low risk of bias.” In addition, we assessed publication bias by using a funnel plot and Egger’s test.

### Meta-analysis

All analyses were conducted in R using toolboxes referenced in the SI4 in the Supplementary Material. A random-effects model with Hartung-Knapp adjustment (Hartung & Knapp, 2001) was calculated. Because different scales were used, outcome variables were re-estimated as the bias-corrected standardized mean difference (Hedges’ *g*) between the treatment and control groups at the first post-treatment assessment using exact formulae. Hereby, negative values indicate a decrease in RNT after therapy compared to the control condition. The stability of the effect was calculated exploratorily as the standardized mean difference values between the CBT group and the control group at the first follow-up assessment in a short-term follow-up period (≤ 6 months). In addition to the effect size estimators, we computed the respective 95% confidence intervals (CI). To determine heterogeneity among the estimated mean effect size, we used *τ^2^*, *I^2,^* and 95% prediction intervals (PI). Sensitivity analysis was conducted to assess the risk of bias, and robustness of effects using a *leave-one-out* (“jackknife”) procedure (Viechtbauer, 2010), and to compare multiple active CBT treatment groups as preregistered. Statistical heterogeneity for sensitivity and subgroup analysis was assessed using *I^2^* and Cochran’s *Q* to determine the degree of between-group heterogeneity that cannot be attributed to sampling error. The significance of subgroup differences was assessed using the *Q*-test. We assumed the within-group heterogeneity of subgroups to be different.

## Results

### Selection and inclusion of studies

**Figure 1.**
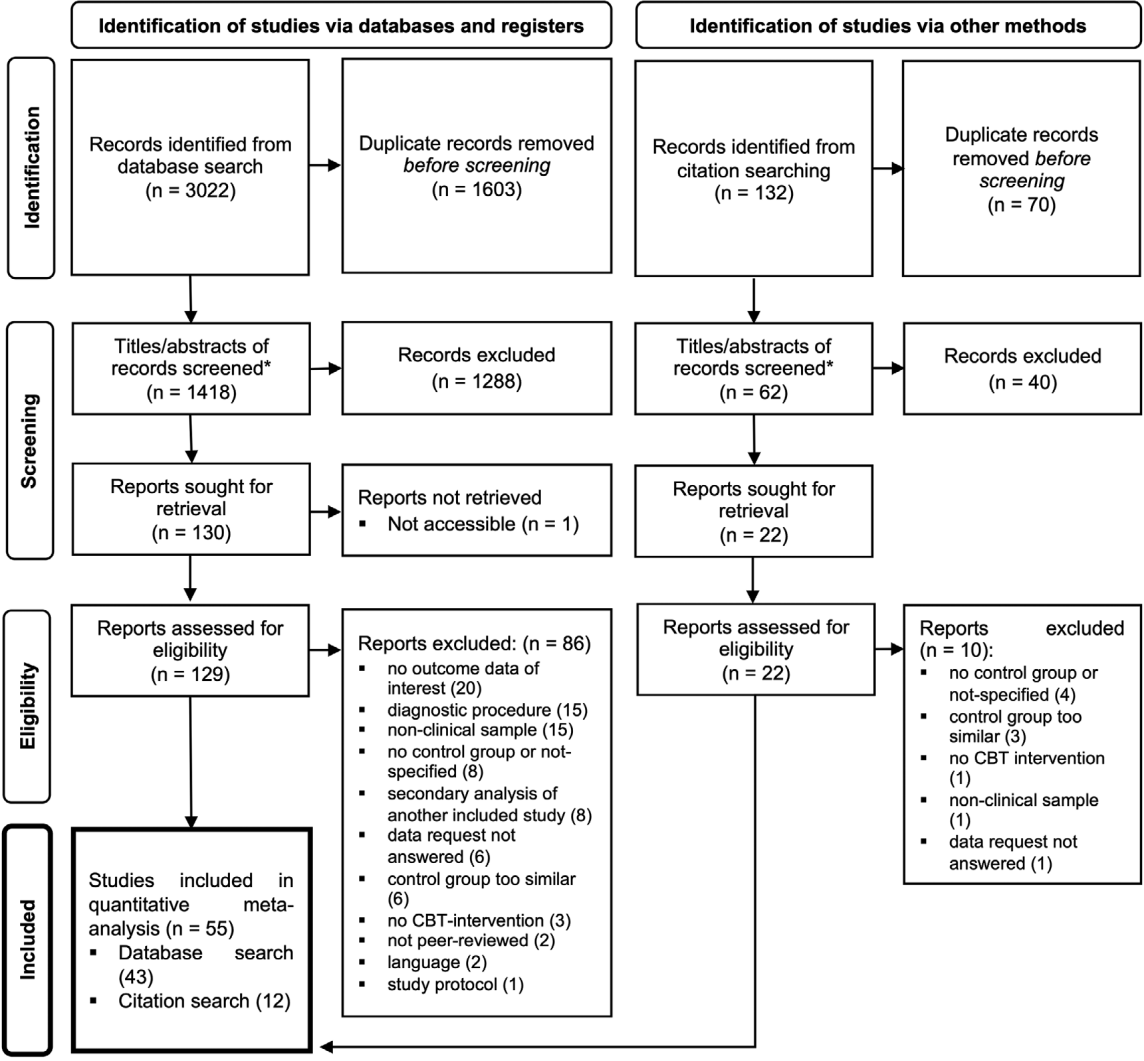
PRISMA flowchart for study selection. *Note*: CBT, cognitive behavioral therapy; *n*, number of individual studies. *, title screening and abstract screening were two separate steps.

The database search yielded a total of 3,022 records. An additional 132 were identified by reviewing reference lists of previous meta-analyses. After duplicate removal, the titles and abstracts of 1,480 records were screened for exclusion criteria independently by two investigators (K.L.S. *and* M.B. *or* J.K.) reaching moderate inter-rater reliability (Cohen’s κ = 0.39). After having reached a full consensus by discussion and having removed non-eligible studies, 152 studies remained. After retrieval of 151 studies, full texts were checked for eligibility (κ = 0.61), and consensus was reached again, resulting in 66 eligible studies. Eleven studies were excluded during data extraction because eligibility criteria were not met (4) or because data requests from the corresponding authors were not successful (7). Thus, a total of 55 studies were used for quantitative analysis (see Figure 1 for details). These yielded 67 comparisons due to multiple treatment arms or control groups within one study.

### Study characteristics

The 55 studies included 4,970 unique participants in total (2,644 in the treatment groups and 2,326 in the control groups). Selected study characteristics of all included studies prior to outlier exclusion can be found in the SI6 in the Supplementary Material (see SI5 in the Supplementary Material for study references and supplementary data on OSF for complete study characteristics). The mean age was 43.31 years (SD = 10.42) and the average proportion of females was 70.9%. Most studies included GAD as a primary diagnosis (35 studies). Other anxiety disorders were also investigated (21 studies), whereas depressive disorders including bipolar were represented less frequently (16 studies). Four studies investigated insomnia disorders. Worry was the most common construct investigated (37 studies), followed by rumination (13 studies), and transdiagnostic RNT (5 studies). Out of 17 RNT-specific treatment groups, the treatment protocols of 14 groups were explicitly worry-specific, only two were transdiagnostic RNT-specific, and only one was rumination-specific. Hence, worry was by far the most targeted construct, followed by transdiagnostic RNT and rumination.

### Overall effect of CBT

**Figure 2.**
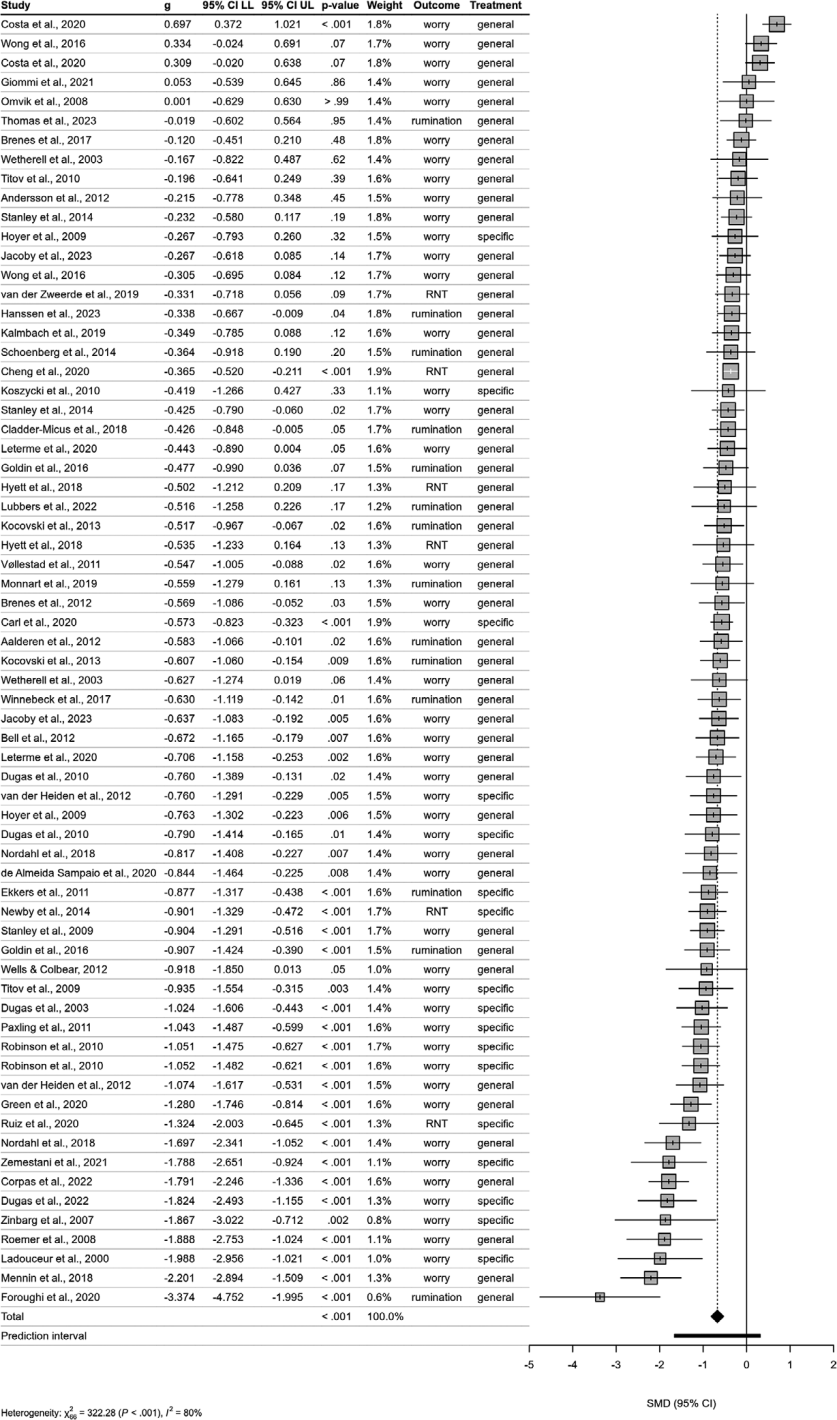
Forest plot of included studies examining the effect of cognitive behavioral therapy compared with control group on repetitive negative thinking at post-treatment. *Note*: Negative values indicate improvement in repetitive negative thinking. The position of the diamond shape indicates the average effect and its width indicates the confidence interval of the pooled result. The horizontal bar indicates the prediction interval – a range into which the effects of future studies may fall based on present evidence. *g*, Hedge’s *g*; CI, confidence interval; LL, lower level; UL, upper level. Treatment, treatment specificity. X^2^, chi-square test of heterogeneity – higher values indicate that observed differences can less likely be explained by chance alone. I^2^, measure of between-study heterogeneity. SMD, Standardized Mean Difference.

Overall, the estimated mean of the true effects of any active CBT treatment in reducing post-intervention RNT compared to any control group was moderate to high in favor of the CBT group (*g* = −0.73, 95% CI: −0.94 to −0.51, *k* = 69). The variance of the distribution of true effect sizes was estimated at *τ^2^* = 0.31 with a wide 95% CI of 0.76 to 3.14. The *I*^2^ estimate of 83.5% (95% CI: 79.7–86.6) indicated substantial between-study heterogeneity (Higgins, Thompson, Deeks, & Altman, 2003). The 95% PI ranged from *g* = −1.86 to 0.40. However, two studies reported extreme effect sizes of *g* = −16.74 (Abdollahi, Hosseinian, Panahipour, & Allen, 2021) and *g* = −2.84 (Nasiri, Mashhadi, Bigdeli, Chamanabad, & Ellard, 2020) and were considered highly influential studies according to the criteria described above. These outliers were excluded from all further analyses as they would have drastically inflated the effect size estimates (for a forest and funnel plot including the outliers, see SI8 and SI9 in the Supplementary Material). After having removed the outliers, the effect of any CBT treatment on post-intervention on any RNT outcome compared to any control group was moderate in favor of the CBT group (*g* = −0.67, 95% CI: −0.82 to −0.53, *k* = 67). After outlier removal, the variance of true effect sizes decreased to *τ^2^* = 0.24 (95% CI: 0.17–0.46). Yet, a substantial *I*^2^ value of 79.5% (95% CI: 74.4–83.6) was preserved. The 95% PI became smaller now ranging from *g* = −1.66 to 0.31 (see Figure 2).

### Effects for different RNT constructs

The efficacy of CBT interventions did not differ substantially between RNT constructs (*p* = .884). Content-independent RNT (*g =* −0.60, 95% CI = −0.98 to −0.22, *I^2^* = 0.59, *k =* 6), rumination (*g =* −0.55, 95% CI = −0.76 to −0.34, *I^2^* = 0.50, *k =* 14), and worry (*g =* −0.70, 95% CI = −0.89 to −0.52, *I^2^* = 0.84, *k =* 47) were addressed equally by CBT interventions. Descriptively, worry was addressed better than RNT, which was addressed better than rumination (see Table 1).

### Quality assessment

**Figure 3.**
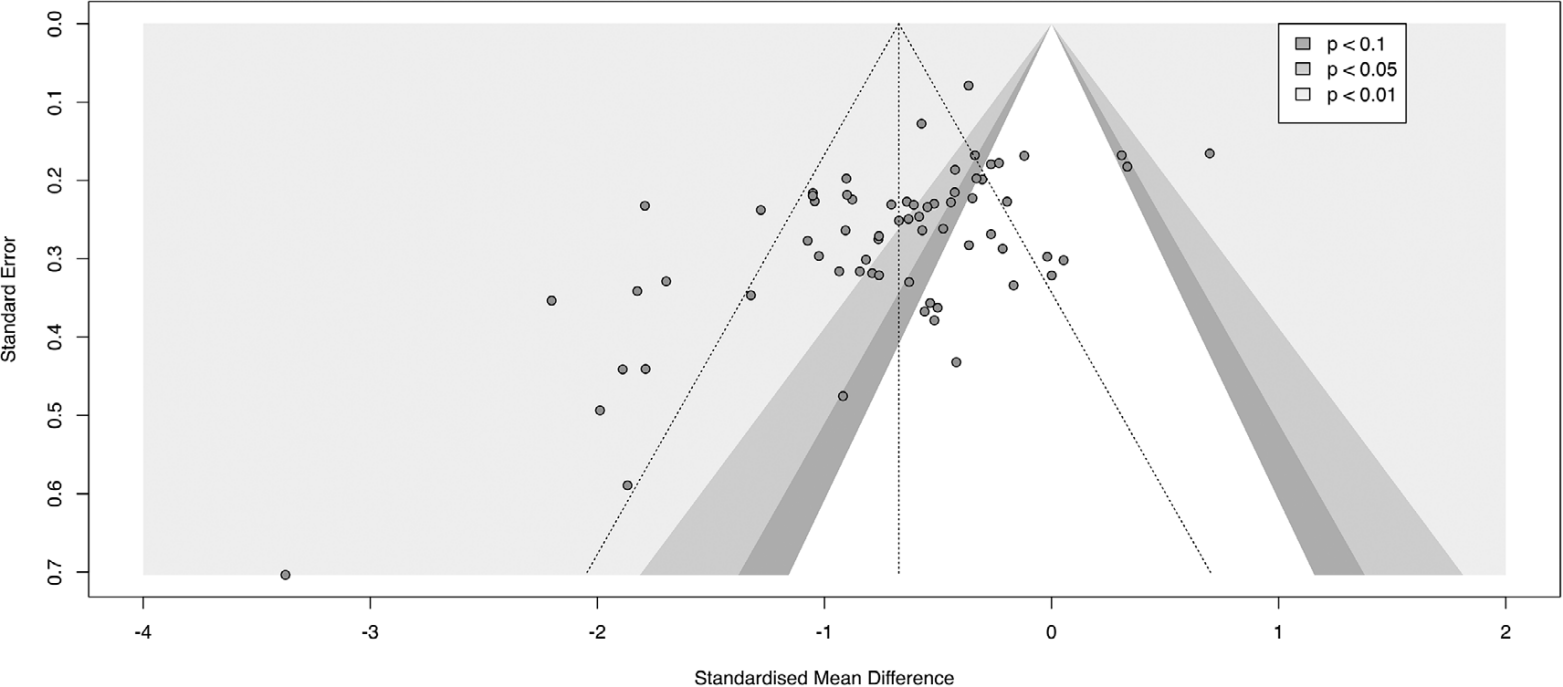
Contour-enhanced Funnel plot of included studies examining the effect of cognitive behavioral therapy compared with control group on repetitive negative thinking at post-treatment.

The quality of the included studies was mixed. 9 studies showed a low risk of bias indicating proper randomized allocation, allocation concealment, blinding, and complete and unselective outcome data report. 25 showed a medium risk of bias indicating proper allocation and concealment, but improper blinding or selective reporting. 21 showed a high risk of bias, indicating unclear or improper allocation and concealment. 30 studies used an intention-to-treat analysis (see SI6 in the Supplementary Material for details). Publication bias was further assessed by using a contour-enhanced funnel plot (Figure 3) suggesting that the beneficial effect of CBT is driven by rather small studies with high standard errors, whereas large studies (e.g., Costa et al., 2020) generally show smaller effects. The Egger’s test was also significant, *t*(66) = 4.39, *p* < .001 suggesting funnel plot asymmetry.

### Sensitivity analyses

After outlier removal, an influence analysis demonstrated that the removal of none of the studies (leave-one-out procedure) changed the overall effect size estimate significantly which indicates a robust effect. Also, the studies’ risk of bias did not influence the overall effect size significantly (*p* = .961). With respect to choosing between multiple treatment groups, the most conservative estimate yielded an effect size of *g* = −0.65, while the most liberal effect size estimate was −0.70 in favor of CBT. With respect to choosing between multiple control groups, the most conservative estimate yielded an effect size of *g* = −0.70, while the most liberal effect size estimate was *g* = −0.72 in favor of CBT (see Table 1 for details).

### Meta-regressions and subgroup analyses

Subgroup analysis suggested a significant differential effect of treatment specificity *Q*(1) = 10.04, *p* = .002, such that general approaches yield smaller effect sizes (*g* = −0.56) than RNT-specific interventions (*g* = −0.99). This effect remained significant in every iteration of a leave-one-out procedure indicating a robust effect. Second, there was no significant differential effect of setting *Q*(1) = 2.80, *p* = .094, such that individual settings did not yield significantly larger effect sizes than group settings. Furthermore, there was no significant differential effect of mode of delivery *Q*(1) = 1.36, *p* = .243, such that in-person settings did not yield larger effect sizes than other delivery modes. Forth, there was a significant differential effect of control type *Q*(1) = 5.36, *p* = .021, indicating that active control group designs yielded significantly smaller effect sizes than passive control group designs. Refer to Table 1 for details.

The number of therapy sessions accounted for 6.87% of heterogeneity in true effect sizes. The test of the regression coefficient was significant (*t*(64) = −2.29, *p* = .025) indicating that for each therapy session, the effect size *g* is expected to increase by 0.04. However, the *Q-*Test for residual heterogeneity was significant (*Q*(64) = 308.79, *p* < .001) suggesting that a substantial amount of heterogeneity was still not accounted for. Also, the upper limit of administered sessions was 20 limiting generalizability. The publication year only accounted for 0.57% of heterogeneity in true effect sizes while the test of the regression coefficient was also not significant (*t*(65) = 0.92, *p* = .363).

At the first follow-up assessment (mean = 4.2 months after post-treatment), the overall effect size was *g* = −0.66 (95% CI: −1.08 to −0.24, *I^2^* = 78.3%, *k* = 16), which does not differ considerably from the overall effect of *g* = −0.67 (95% CI: −0.82 to −0.53, *I^2^* = 79.5%) found at post-treatment.

## Discussion

This meta-analysis investigated the transdiagnostic efficacy of CBT interventions on RNT and its subconstructs worry and rumination. We explored which aspect of RNT is treated best by current CBT interventions. Furthermore, we studied the differential effects of RNT-specific treatments. Independent of the RNT subconstruct investigated, CBT interventions had a moderate overall efficacy in reducing RNT at post-treatment compared to control groups. RNT-specific therapies were significantly more efficacious than more general approaches. The post-treatment effect size was moderate when the control group was active, but significantly different and large when the control group was passive. The number of sessions administered significantly increased the effect size within a 20-session limit. The overall effect size at the first follow-up assessment was similar to the effect size at post-treatment suggesting a short-term stability of therapy effects.

### Overall efficacy

The overall treatment efficacy of CBT in reducing RNT in this study (*g* = −0.67) was comparable, but slightly smaller than estimates of general symptom reduction by CBT for MDD (*g* = −0.75) or GAD (*g* = −0.80) in a previous meta-analysis (Cuijpers, Cristea, Karyotaki, Reijnders, & Huibers, 2016). This indicates that RNT is generally addressed moderately well by CBT, even without being the primary target of intervention. This is in line with previous findings suggesting that RNT mediates symptom improvement for depression and anxiety (Spinhoven, Klein, et al., 2018; Spinhoven, Van Hemert, et al., 2018). Nevertheless, CBT interventions should be improved to better address RNT.

Previous meta-analyses investigating the effect of psychotherapy on RNT generally found smaller overall effects of different therapeutic approaches on RNT. We propose that differences in the studied populations and treatments in previous work compared to our analysis could contribute to the observed variations in outcomes: Compared to Spinhoven, Klein, et al. (2018) focusing on CBT for depression, the overall effect estimates of CBT in reducing RNT were slightly larger in our study (*Δg* = 0.19). The authors included non-clinical subjects and may have estimated a smaller improvement due to floor effects. Also, the study focused on depression-focused CBT. It is known that psychotherapy for depression generally shows smaller effects than for anxiety disorders (Cuijpers et al., 2016), so that the difference may also be partly explainable by the conditions that were studied. This might also explain why the difference to Monteregge et al. (2020) who focused exclusively on anxiety disorders, was less pronounced (*Δg* = −0.01), although they included subclinical populations and therapies other than CBT. Compared to Bell, Marx, et al. (2023), our effect size was probably larger (*Δg* = 0.22) due to the fact that their sample comprised more subclinical than clinical subjects. Also, their sample more often received mindfulness training and e.g., less often the more effective worry exposure treatments compared to our sample. It is important to emphasize that while these choices led to different overall results, they all contribute to a more nuanced understanding of the effects of various forms of psychotherapy on RNT. In particular, including subclinical populations (Bell, Marx, et al., 2023; Spinhoven, Klein, et al., 2018) acknowledges the transdiagnostic conceptualization of RNT, while constraining the mental disorders or treatment included allows the investigation of possible treatment mechanisms (Spinhoven, Klein, et al., 2018). To complement the groundwork set by these studies, we opted for a comprehensive clinical meta-analysis, including subjects with various mental disorders and a diverse range of CBT interventions (e.g., ACT, exposure therapies, emotion regulation therapy, MBCT, MCT, Unified Protocol). For a clinical population, we found that most CBT interventions are effective in improving RNT, while some are even highly effective, such as MBCT (Foroughi et al., 2020), emotion regulation therapy (Mennin, Fresco, O’Toole, & Heimberg, 2018), a worry program involving intolerance of uncertainty (Robert Ladouceur et al., 2000), and Acceptance-Based Behavior Therapy (Roemer, Orsillo, & Salters-Pedneault, 2008).

The prediction interval for the overall effect contained effect sizes indicating larger post-intervention RNT values in the CBT groups compared to control groups in a minority of studies. Therefore, adverse treatment effects of CBT on RNT might not be fully ruled out. Nevertheless, the likelihood of adverse treatment effects can be considered rather low, as only two studies suggested adverse effects, where of one study (Costa et al., 2020) investigated a newly developed mindfulness intervention.

### Efficacy for different RNT constructs

Our analysis suggests that all RNT constructs were addressed equally by CBT, which is in line with previous meta-analytic findings of Spinhoven, Klein, et al. (2018). Descriptively, treatment efficacy was largest for worry and smallest for rumination. This is not consistent with previous findings, but is well explained by the fact that they used depression-focused CBT, which tends to focus on rumination rather than worry. The descriptive trend in our study may be explained by the fact that most RNT-specific interventions, for which treatment effects were significantly larger, addressed worry. Generally, worry was the most investigated construct in our sample being the cardinal symptom of GAD, for which treatment effectiveness is large (*g* = −0.80; Cuijpers et al., 2016). Nevertheless, the fact that no RNT subconstruct was addressed significantly better or worse may underscore a process-focused apprehension of RNT as suggested earlier (Bell, Marx, et al., 2023; Rosenkranz et al., 2020).

### Efficacy of RNT-specific interventions

RNT-specific interventions seemed to outperform general approaches significantly (*Δg* = 0.43). This challenges the findings of previous meta-analyses by Monteregge et al. (2020) and Bell, Marx, et al. (2023) suggesting that RNT-focused and non-RNT-focused psychological interventions did not differ significantly. In contrast to these studies, we opted for a “lexical” approach to study classification: Two study authors independently screened the original studies for explicit statements describing the aim to address an RNT construct by the intervention. Consequently, unlike previous reviews, we did not include MCT, mindfulness-based therapies, training, and interventions, behavioral activation, and emotion regulation therapy as RNT-focused treatments by default. While the proposed mechanisms of change of each of these interventions make it valid to consider them RNT-focused — MCT and mindfulness seek to change meta-cognitions potentially contributing to RNT, behavioral activation, and emotion regulation foster adaptive coping to counter RNT, and general CBT challenges negative thought content — some do not explicitly target RNT or address multiple or ambiguous processes. We instead prioritized acknowledging a given study’s explicit treatment purpose as RNT-specific or not, rather than comparing complex treatment packages (Rief et al., 2024). Although our approach is also an approximation to a more complex truth, the results suggest that using a more fine-grained, study-by-study coding process provides evidence pointing towards the superiority of RNT-targeted treatments compared to other types of CBT.

The significant difference is also not surprising since most interventions for RNT in our study focused on treating pathological worry, which usually involves effective, transdiagnostic interventions, such as worry exposure (van Dis et al., 2020). The interaction of a selective sampling of this circumscribed population with a well-understood disorder process, and a highly effective treatment may have increased the effect size difference in our study. One should generally bear in mind that the advantage of specific treatments is most pronounced when the symptoms are primarily caused by the targeted transdiagnostic factor, such as worry (the cardinal symptom of GAD) being addressed by specialized GAD treatments (Olatunji et al., 2013).

This finding underscores the need for the development and dissemination of already existing treatments targeting RNT. Since most specific treatments effectively address worry, we especially advocate the broader application of rumination and transdiagnostic RNT-focused treatments. Rumination-focused CBT (Watkins, 2018) may pose such a promising treatment option encompassing well-known active ingredients of CBT, such as behavioral activation, functional analysis, and cognitive restructuring which are tailored to rumination. So far, few RCTs have used these protocols. Based on the presented findings we assume that such interventions could address RNT in a more efficacious way than targeting the process indirectly with non-specialized treatment.

### Limitations

First, by emphasizing individual study descriptions over overarching treatment rationales when classifying studies as “RNT-focused,” we may have inadvertently generated “false negative” classifications (i.e., overlooking studies specifically targeting RNT). This could have resulted in lower sensitivity in identifying RNT-focused treatments, as a study was only included as “specific” when the study’s authors explicitly stated that the therapeutic approach specifically targeted RNT. Nevertheless, we believe that acknowledging the study authors’ treatment classification approach prevents interventions derived from manifold theories from being classified as targeting a specific process by default. However, concerns about false negatives should not be overestimated, as our use of random effect models in estimating effect sizes allows for the generalization of findings to unconsidered studies that meet the inclusion criteria (Borenstein, Hedges, Higgins, & Rothstein, 2009).

Second, the obtained effect sizes were not always independent due to the use of multiple treatment or control groups for effect size calculations in case of multiple active treatments or multiple control groups (*unit-of-analysis error*, Rücker et al., 2017). However, we decided not to let our degrees of freedom bias the effect size. Instead, we opted for reporting the results of a “multiverse” sensitivity analysis providing possible alternatives demonstrating that none of these choices biased the effect size substantially suggesting that the procedure may also have led to a more unbiased estimate since treatment and control groups with higher and lower effect sizes contributed to the overall effect size. Yet, this procedure may have led to an inaccurate estimate of variance for the overall effect potentially inflating the influence of some multi-arm studies.

Third, allegiance effects cannot be ruled out, as some study authors and therapists were also the authors of the treatment protocols used (Ladouceur et al., 2000; Mennin, Fresco, O’Toole, & Heimberg, 2018; Nordahl et al., 2018; Wells & Colbear, 2012). In addition, these studies uniformly used passive control group designs. Furthermore, multiple indicators of selective publication were found (i.e., funnel-plot asymmetry, significant Egger’s test). These issues might have led to an overestimation of the reported effects.

Lastly, subgroups were small and analyses might be underpowered. Hence, the moderation effects should be interpreted with caution, particularly if effect size differences are marginal, subgroups are small, and/or have a low between-study heterogeneity. Significant moderation should generally be interpreted as a correlational rather than a causal finding (Hedges & Pigott, 2001) and experimental evidence would be required to corroborate them (Rott et al., 2024).

### Future directions

RNT-focused CBT was found to be highly efficacious in reducing RNT. However, other forms of CBT only demonstrated a moderate reduction of RNT. Thus, future studies should find out which treatment works best for whom under which conditions (Paul, 1967). First, it is important to find out what the “active ingredients” are by deploying mechanistic studies to better understand general maladaptive processes of RNT (e.g., possible links to anhedonia; Rutherford, McDougle, & Joormann, 2023), by testing individual treatment components using appropriate designs to detect “active ingredients” (Blackwell, 2024), and by leveraging computational modeling to enable more accurate predictions and model testing (Bedder, Pisupati, & Niv, 2023; Berg, Feldmann, Kirchner, & Kube, 2022; Robinaugh, Haslbeck, Ryan, Fried, & Waldorp, 2021). Second, it is equally important to consider individual differences by conducting naturalistic studies (e.g., using ecological sampling methods and network modeling) to better understand individual symptom dynamics (Berg et al., 2022; Rosenkranz et al., 2020; Westhoff, Berg, Reif, Rief, & Hofmann, 2024) to facilitate and inform individualized treatment (Bell, Arnold, et al., 2023). Furthermore, it is imperative to use scalable approaches to deliver these effective components to a wide population (Bell, Arnold, et al., 2023; Funk, Kopf-Beck, Watkins, & Ehring, 2023).

Currently, although promising treatments targeting RNT are available (e.g., rumination-focused CBT, and internet-based worry programs), they have rarely been used in RCTs. Hence, when clinical psychology seeks to establish RNT, it needs to investigate, implement, and disseminate RNT-focused interventions more thoroughly.

### Summary

This meta-analysis investigated the transdiagnostic efficacy of CBT treatments across different RNT constructs. It showed that the efficacy of CBT treatments on RNT is generally moderate and suggests that intervention effects can be enhanced substantially by using specialized RNT treatments. Hence, specialized, RNT-focused treatments should be investigated, implemented, and disseminated more rigorously.

## Supporting information

Stenzel et al. supplementary material Stenzel et al. supplementary material

## Supplementary material

The supplementary file for this article can be found at http://doi.org/10.1017/S0033291725000017.
