# Efficacy of cognitive behavioral therapy in treating repetitive negative thinking, rumination, and worry – a transdiagnostic meta-analysis – CORRIGENDUM

**DOI:** 10.1017/S0033291726103304

**Published:** 2026-01-28

**Authors:** Kilian Leander Stenzel, Joshua Keller, Lukas Kirchner, Winfried Rief, Max Berg

**Affiliations:** 1 Department of Psychology, Division of Clinical Psychology and Psychotherapy, Marburg University, Marburg, Germany; 2 Clinical Psychology and Psychotherapy, Justus-Liebig-University Giessen, Giessen, Germany

In response to Bailey (2025), we revisited the subgroup classification of the *treatment specificity* variable, that is, whether a treatment was considered *specialized* for repetitive negative thinking (RNT) or *general.* We considered the critique of a potential misclassification of treatments and repeated our classification procedure arriving at a plausibly more consistent solution. Specifically, we reassigned meta-cognitive therapy treatments, Applied Relaxation treatments, and one Emotion Regulation Therapy treatment to the *specialized* (RNT-specific) group. For a more detailed discussion, please refer to our response letter (Stenzel et al., 2026) to the letter by Bailey (2025). Most importantly, no conclusions were affected by this. However, after reclassifying the treatments and the reanalysis, the following statistical values (in bold) have changed and should be substituted. Also, the Table 1 and the Figure 2 have changed and should be updated.

**p. 1: Abstract**

RNT-specific interventions (***g* = −0.87**) were significantly more efficacious in reducing RNT than less specific approaches (***g* = −0.48**).

**p. 6: Meta-regressions and subgroup analyses**

Subgroup analysis suggested a significant differential effect of treatment specificity ***Q*(1) = 8.05**, ***p* = 0.005**, such that general approaches yield smaller effect sizes (***g* = −0.48**) than RNT-specific interventions (***g* = −0.87**).

**p. 9: Efficacy of RNT-specific interventions**

RNT-specific interventions seemed to outperform general approaches significantly (***Δg* = 0.39**).

**p. 4:**

**Table 1.** Effects of cognitive behavioral therapy on repetitive negative thinking compared with control groups at post-treatment

Variable	Levels	*k^a^*	*g^b^*	95% CI	*I^2^* (%)	*p^c^*
Overall effect		67	−0.67	[−0.82; −0.53]	80	
Sensitivity analyses						
Multiple treatments	Smaller effect – conservative	57	−0.65	[−0.81; −0.48]	81	
	Larger effect – liberal	57	−0.70	[−0.87; −0.53]	82	
Multiple controls	Smaller effect – conservative	63	−0.70	[−0.85; −0.55]	79	
	Larger effect – liberal	63	−0.72	[−0.86; −0.58]	74	
Risk of bias	High	26	−0.68	[−0.89; −0.47]	58	0.961
	Medium	30	−0.65	[−0.90; −0.40]	87	
	Low	11	−0.65	[−0.89; −0.39]	57	
Outcome	RNT	6	−0.60	[−0.98; −0.22]	59	0.522
	Rumination	14	−0.55	[−0.76; −0.34]	50	
	Worry	47	−0.70	[−0.89; −0.52]	84	
Subgroup analyses						
**Treatment specificity**	**General**	**33**	**−0.48**	**[−0.69; −0.28]**	**81**	0.005*
	**Specialized**	**34**	**−0.87**	**[−1.06; −0.68]**	**67**	
Setting A	Group	28	−0.53	[−0.77; −0.28]	84	0.094
	Individual	39	−0.77	[−0.95; −0.60]	73	
Setting B	In-person	50	−0.72	[−0.91; −0.52]	83	0.243
	Other^d^	17	−0.57	[−0.74; −0.0]	60	
Type of control group	Active	27	−0.46	[−0.73; −0.20]	84	0.021*
	Passive	40	−0.81	[−0.96; −0.65]	64	

*Note*: ^a^*k* indicates the number of comparisons for each level. ^b^*g* indicates Hedge’s *g.* ^c^*p* indicates whether the effect sizes of subgroups differed significantly from each other based on a *Q*-test. ^d^Other indicates internet-based therapy with or without in-person support, phone call, video call, or mixed therapy (in-person and phone call).

* *p <* 0.05.

**p. 7:**

**Figure 2.**
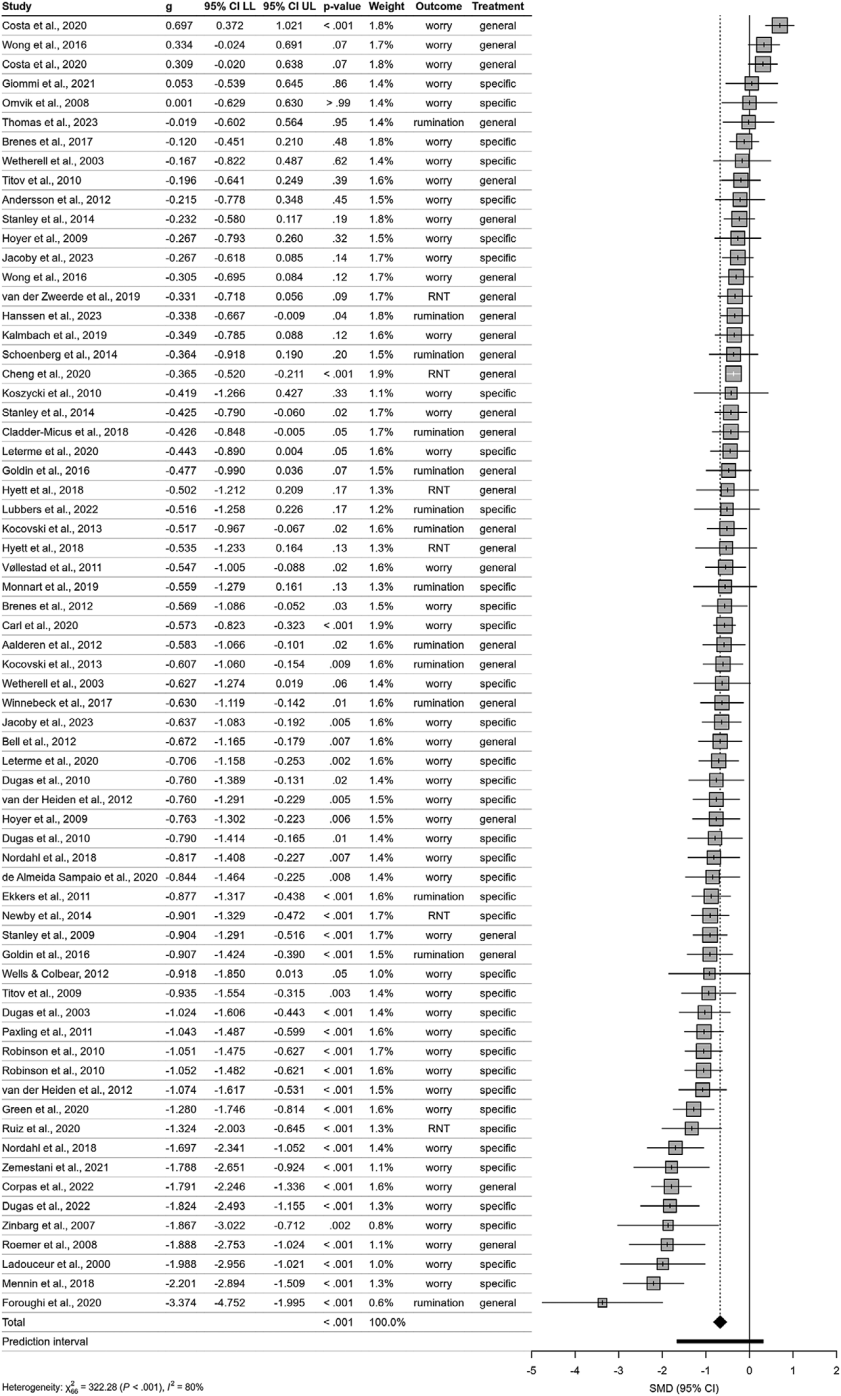
Forest plot of included studies examining the effect of cognitive behavioral therapy compared with control group on repetitive negative thinking (RNT) at post-treatment. *Note*: Negative values indicate improvement in RNT. The position of the diamond shape indicates the average effect and its width indicates the confidence interval of the pooled result. The horizontal bar indicates the prediction interval – a range into which the effects of future studies may fall based on present evidence. *g*, Hedge’s *g*; CI, confidence interval; LL, lower level; UL, upper level; Treatment, treatment specificity with regard to RNT; X^2^, chi-square test of heterogeneity – higher values indicate that observed differences can less likely be explained by chance alone; *I* ^2^, measure of between-study heterogeneity; SMD, Standardized Mean Difference.
