# Supplementary material for: Efficacy of cognitive behavioral therapy in treating repetitive negative thinking, rumination, and worry – a transdiagnostic meta-analysis
Source: Psychol Med. 2025 Feb 7;55:e31. doi: 10.1017/S0033291725000017 (PMC12017360; doi:10.1017/S0033291725000017)
Supplement: Stenzel et al. supplementary material [file S0033291725000017sup001.docx]

**Supplementary Information for the Paper: “Efficacy of Cognitive Behavioral Therapy in Treating Repetitive Negative Thinking, Rumination, and Worry - A Transdiagnostic Meta-analysis.”**

by

Kilian Stenzel et al.

**SI1:** Final list of search strings applied for databases

The search strategy was identical for all five databases apart from differences in controlled vocabulary which was adapted to the respective database. For example, the following search string was applied to EMBASE:

(‘psychosexual disorder’:ab,kw,ti OR ‘sexual dysfunction’:ab,kw,ti OR ‘sexual problems’:ab,kw,ti OR ‘eating disorder’:ab,kw,ti OR ‘anorexia nervosa’:ab,kw,ti OR ‘anorexia‘:ab,kw,ti OR ‘binge eating‘:ab,kw,ti OR ‘binge eating disorder’:ab,kw,ti OR ‘bulimia’:ab,kw,ti OR ‘eating pathology’:ab,kw,ti OR ‘fear’:ab,kw,ti OR ‘anxiety disorder’:ab,kw,ti OR ‘anxiety‘:ab,kw,ti OR ‘obsessive compulsive disorder’:ab,kw,ti OR ‘OCD‘:ab,kw,ti OR ‘social anxiety’:ab,kw,ti OR ‘social phobia’:ab,kw,ti OR ‘SAD‘:ab,kw,ti OR ‘Panic Disorder‘:ab,kw,ti OR ‘panic’:ab,kw,ti OR ‘PD’:ab,kw,ti OR ‘phobia‘:ab,kw,ti OR ‘agoraphobia‘:ab,kw,ti OR ‘generalized anxiety disorder‘:ab,kw,ti OR ‘generalised anxiety disorder‘:ab,kw,ti OR ‘GAD‘:ab,kw,ti OR ‘posttraumatic stress disorder‘:ab,kw,ti OR ‘post-traumatic’:ab,kw,ti OR ‘PTSD‘:ab,kw,ti OR ‘cluster B personality disorder’:ab,kw,ti OR ‘Borderline personality disorder’:ab,kw,ti OR ‘borderline state’:ab,kw,ti OR ‘Borderline’:ab,kw,ti OR ‘BPD’:ab,kw,ti OR ‘mood disorder’:ab,kw,ti OR ‘depressive disorder’:ab,kw,ti OR ‘depression’:ab,kw,ti OR ‘affective disorder’:ab,kw,ti OR ‘affective’:ab,kw,ti OR ‘depressed’:ab,kw,ti OR ‘depressive’:ab,kw,ti OR ‘major depression’:ab,kw,ti OR ‘MDD’:ab,kw,ti OR ‘dysthymia‘:ab,kw,ti OR ‘dysthymic disorder’:ab,kw,ti OR ‘bipolar disorder‘:ab,kw,ti OR ‘bipolar‘:ab,kw,ti OR ‘bipolar I disorder’:ab,kw,ti OR ‘bipolar II disorder’:ab,kw,ti OR ‘mania’:ab,kw,ti OR ‘manic’:ab,kw,ti OR ‘schizophrenia’:ab,kw,ti OR ‘schizophrenia spectrum disorder’:ab,kw,ti OR ‘psychosis’:ab,kw,ti OR ‘psychotic’:ab,kw,ti OR ‘psychotic mood disorder’:ab,kw,ti OR ‘schizotypal personality disorder’:ab,kw,ti OR ‘schizoid personality disorder’:ab,kw,ti OR ‘paranoid personality disorder’:ab,kw,ti OR ‘substance abuse’:ab,kw,ti OR ‘substance-related disorders’:ab,kw,ti OR ‘substance use disorder’:ab,kw,ti OR ‘addiction’:ab,kw,ti OR ‘drug abuse’:ab,kw,ti OR ‘drug use’:ab,kw,ti or ‘alcoholism’:ab,kw,ti OR ‘drug dependence’:ab,kw,ti OR ‘drug misuse’:ab,kw,ti OR ‘antisocial personality disorder’:ab,kw,ti OR ‘conduct disorder’:ab,kw,ti OR ‘oppositional defiant disorder’:ab,kw,ti OR ‘ADHD’:ab,kw,ti OR ‘attention deficit hyperactivity disorder’:ab,kw,ti OR ‘intermittent explosive disorder’:ab,kw,ti OR ‘narcissistic personality disorder’:ab,kw,ti OR ‘histrionic personality disorder’:ab,kw,ti OR ‘paranoid personality disorder’:ab,kw,ti OR ‘avoidant personality disorder’:ab,kw,ti OR ‘dependent personality disorder’:ab,kw,ti OR ‘histrionic personality disorder’:ab,kw,ti) AND (‘cognitive behavioral therapy’:ab,kw,ti OR ‘cognitive behaviour therapy’:ab,kw,ti OR ‘cognitive behavior therapy’:ab,kw,ti OR ‘CBT’:ab,kw,ti OR ‘cognitive therapy‘:ab,kw,ti OR ‘cognitive treatment’:ab,kw,ti OR ‘cognitive intervention’:ab,kw,ti OR ‘Thirdwave therapies’:ab,kw,ti OR ‘Third wave cognitive behavioural therapies’:ab,kw,ti OR ‘acceptance and commitment therapy’:ab,kw,ti OR ‘ACT’:ab,kw,ti OR ‘mindfulness-based cognitive therapy’:ab,kw,ti OR ‘MBCT’:ab,kw,ti OR ‘mindfulness-based stress reduction’:ab,kw,ti OR ‘MBSR’:ab,kw,ti OR ‘rumination-focused’:ab,kw,ti OR ‘RFCBT’:ab,kw,ti OR ‘Metacognitive Therapy’:ab,kw,ti OR ‘MCT’:ab,kw,ti OR ‘dialectical behavior therapy’:ab,kw,ti OR ‘DBT’:ab,kw,ti OR ‘schema therapy’:ab,kw,ti OR ‘CBASP’:ab,kw,ti) AND (‘ruminat*’:ab,kw,ti OR ‘cognitive rumination’:ab,kw,ti OR ‘repetitive thought’:ab,kw,ti OR ‘repetitive thinking’:ab,kw,ti OR ‘recurrent thought’:ab,kw,ti OR ‘repetitive negative thinking’:ab,kw,ti OR ‘repetitive negative thought’:ab,kw,ti OR ‘RNT’:ab,kw,ti OR ‘worry’:ab,kw,ti OR ‘worrying’:ab,kw,ti OR persev*:ab,kw,ti OR ‘intrusive thought’:ab,kw,ti OR ‘intrusive thinking’:ab,kw,ti OR ‘negative thought’:ab,kw,ti OR ‘negative thinking’:ab,kw,ti OR ‘stress thought’:ab,kw,ti OR ‘stress thinking’:ab,kw,ti OR ‘obsessive thought’:ab,kw,ti OR ‘obsessive thinking’:ab,kw,ti OR ‘unconscious stress’:ab,kw,ti OR ‘implicit stress’:ab,kw,ti OR ‘anticipatory stress’:ab,kw,ti OR ‘anticipation stress’:ab,kw,ti OR ‘cognitive intrusion*’:ab,kw,ti OR reflection:ab,kw,ti OR brooding:ab,kw,ti OR ‘reflect*’:ab,kw,ti OR ‘self referential thought’:ab,kw,ti OR ‘counterfactual thinking’:ab,kw,ti OR ‘mind wandering’:ab,kw,ti OR ‘post-event processing’:ab,kw,ti OR ‘habitual negative self-thinking’:ab,kw,ti OR ‘catastrophizing’:ab,kw,ti OR ‘automatic thoughts’:ab,kw,ti OR ‘everyday thought’:ab,kw,ti OR metacogn*:ab,kw,ti) AND (‘controlled clinical trial’:ab,kw,ti OR ‘randomized controlled trial’:ab,kw,ti OR ‘randomised controlled trial’:ab,kw,ti OR ‘RCT’:ab,kw,ti OR ‘randomized’:ab,kw,ti OR ‘randomised’:ab,kw,ti) AND ([english]/lim OR [german]/lim) AND ([adult]/lim OR [aged]/lim) AND [humans]/lim

**SI2:** Reviews screened to expand study pool

1. Bell, I. H., Marx, W., Nguyen, K., Grace, S., Gleeson, J., & Alvarez-Jimenez, M. (2023). The effect of psychological treatment on repetitive negative thinking in youth depression and anxiety: A meta-analysis and meta-regression. *Psychological Medicine*, *53*(1), 6–16. https://doi.org/10.1017/S0033291722003373
2. Covin, R., Ouimet, A. J., Seeds, P. M., & Dozois, D. J. A. (2008). A meta-analysis of CBT for pathological worry among clients with GAD. *Journal of Anxiety Disorders*, *22*(1), 108–116. https://doi.org/10.1016/j.janxdis.2007.01.002
3. Hall, J., Kellett, S., Berrios, R., Bains, M. K., & Scott, S. (2016). Efficacy of Cognitive Behavioral Therapy for Generalized Anxiety Disorder in Older Adults: Systematic Review, Meta-Analysis, and Meta-Regression. *The American Journal of Geriatric Psychiatry*, *24*(11), 1063–1073. https://doi.org/10.1016/j.jagp.2016.06.006
4. Hanrahan, F., Field, A. P., Jones, F. W., & Davey, G. C. L. (2013). A meta-analysis of cognitive therapy for worry in generalized anxiety disorder. *Clinical Psychology Review*, *33*(1), 120–132. https://doi.org/10.1016/j.cpr.2012.10.008
5. Li, P., Mao, L., Hu, M., Lu, Z., Yuan, X., Zhang, Y., & Hu, Z. (2022). Mindfulness on Rumination in Patients with Depressive Disorder: A Systematic Review and Meta-Analysis of Randomized Controlled Trials. *Int. J. Environ. Res. Public Health*. https://doi.org/10.3390/ijerph192316101
6. Mao, L., Li, P., Wu, Y., Luo, L., & Hu, M. (2023). The effectiveness of mindfulness-based interventions for ruminative thinking: A systematic review and meta-analysis of randomized controlled trials. *Journal of Affective Disorders*, *321*, 83–95. https://doi.org/10.1016/j.jad.2022.10.022
7. Monteregge, S., Tsagkalidou, A., Cuijpers, P., & Spinhoven, P. (2020). The effects of different types of treatment for anxiety on repetitive negative thinking: A meta‐analysis. *Clinical Psychology: Science and Practice*, *27*(2). https://doi.org/10.1111/cpsp.12316
8. Olatunji, B. O., Davis, M. L., Powers, M. B., & Smits, J. A. J. (2013). Cognitive-behavioral therapy for obsessive-compulsive disorder: A meta-analysis of treatment outcome and moderators. *Journal of Psychiatric Research*, *47*(1), 33–41. https://doi.org/10.1016/j.jpsychires.2012.08.020
9. Perestelo-Perez, L., Barraca, J., Peñate, W., Rivero-Santana, A., & Alvarez-Perez, Y. (2017). Mindfulness-based interventions for the treatment of depressive rumination: Systematic review and meta-analysis. *International Journal of Clinical and Health Psychology*, *17*(3), 282–295. https://doi.org/10.1016/j.ijchp.2017.07.004
10. Spinhoven, P., Klein, N., Kennis, M., Cramer, A. O. J., Siegle, G., Cuijpers, P., Ormel, J., Hollon, S. D., & Bockting, C. L. (2018). The effects of cognitive-behavior therapy for depression on repetitive negative thinking: A meta-analysis. *Behaviour Research and Therapy*, *106*, 71–85. https://doi.org/10.1016/j.brat.2018.04.002

**SI3:** Full list of outcome measures and references included in the search

We considered transdiagnostic RNT as measured by the following instruments: (1) Perseverative Thinking Questionnaire (PTQ; Ehring et al., 2011) and (2) the Repetitive Thinking Questionnaire (RTQ) as well as its short version (RTQ-10; McEvoy et al., 2010).

We considered rumination as measured by the following instruments: (1) Ruminative Response Scale (RRS) from the Response Styles Questionnaire (RSQ; Nolen-Hoeksema & Morrow, 1991) and derived therefrom: RRS-10 (Treynor, Gonzalez, & Nolen-Hoeksema, 2003), RRS-8 (Armey et al., 2009), and Response Styles Questionnaire (RSQ-D; Kühner et al., 2007), (2) Rumination-Reflection Questionnaire (RRQ; Trapnell & Campbell, 1999), (3) Rumination on Sadness Scale (RSS; Conway et al., 2000), (4) Post Event Processing Questionnaire (PEPQ; Rachman et al., 2000) and derived therefrom: PEPQ-R (McEvoy & Kingsep, 2006), PEPQ-7 (Kocovski & Rector, 2007), and E-PEPQ (Fehm, Hoyer, Schneider, Lindemann, & Klusmann, 2008).

We considered worry as measured by the following instruments: (1) Penn State Worry Questionnaire (PSWQ; Meyer et al., 1990) and derived therefrom: PSWQ-11 (Hazlett-Stevens, Ullman, & Craske, 2004), PSWQ-Past Week (Stöber & Bittencourt, 1998), and PSWQ-Past Day (Joos et al., 2012), (2) worry subscale of the Thought Control Questionnaire (TCQ; Wells & Davies, 1994), (3) Worry Domains Questionnaire (WDQ; Tallis et al., 1992), as well as the aWDQ (McCarthy-Larzelere et al., 2001), (4) Anxious Thoughts Inventory (ATI; Wells, 1994).

Armey, M. F., Fresco, D. M., Moore, M. T., Mennin, D. S., Turk, C. L., Heimberg, R. G., … Alloy, L. B. (2009). Brooding and Pondering: Isolating the Active Ingredients of Depressive Rumination With Exploratory Factor Analysis and Structural Equation Modeling. *Assessment*, *16*(4), 315–327. https://doi.org/10.1177/1073191109340388

Conway, M., Csank, P. A. R., Holm, S. L., & Blake, C. K. (2000). On Assessing Individual Differences in Rumination on Sadness. *Journal of Personality Assessment*, *75*(3), 404–425. https://doi.org/10.1207/S15327752JPA7503_04

Ehring, T., Zetsche, U., Weidacker, K., Wahl, K., Schönfeld, S., & Ehlers, A. (2011). The Perseverative Thinking Questionnaire (PTQ): Validation of a content-independent measure of repetitive negative thinking. *Journal of Behavior Therapy and Experimental Psychiatry*, *42*(2), 225–232. https://doi.org/10.1016/j.jbtep.2010.12.003

Fehm, L., Hoyer, J., Schneider, G., Lindemann, C., & Klusmann, U. (2008). Assessing post-event processing after social situations: A measure based on the cognitive model for social phobia. *Anxiety, Stress, & Coping*, *21*(2), 129–142. https://doi.org/10.1080/10615800701424672

Hazlett-Stevens, H., Ullman, J. B., & Craske, M. G. (2004). Factor Structure of the Penn State Worry Questionnaire: Examination of a Method Factor. *Assessment*, *11*(4), 361–370. https://doi.org/10.1177/1073191104269872

Joos, E., Vansteenwegen, D., Brunfaut, E., Bastiaens, T., Demyttenaere, K., Pieters, G., & Hermans, D. (2012). The Penn State Worry Questionnaire—Past Day: Development and Validation of a Measure Assessing Daily Levels of Worry. *Journal of Psychopathology and Behavioral Assessment*, *34*(1), 35–47. https://doi.org/10.1007/s10862-011-9265-2

Kocovski, N. L., & Rector, N. A. (2007). Predictors of Post‐Event Rumination Related to Social Anxiety. *Cognitive Behaviour Therapy*, *36*(2), 112–122. https://doi.org/10.1080/16506070701232090

Kühner, C., Huffziger, S., & Nolen-Hoeksema, S. (2007). *Response styles questionnaire: RSQ-D*. Hogrefe.

McEvoy, P. M., & Kingsep, P. (2006). The post-event processing questionnaire in a clinical sample with social phobia. *Behaviour Research and Therapy*, *44*(11), 1689–1697. https://doi.org/10.1016/j.brat.2005.12.005

McEvoy, P. M., Mahoney, A. E. J., & Moulds, M. L. (2010). Are worry, rumination, and post-event processing one and the same? *Journal of Anxiety Disorders*, *24*(5), 509–519. https://doi.org/10.1016/j.janxdis.2010.03.008

Meyer, T. J., Miller, M. L., Metzger, R. L., & Borkovec, T. D. (1990). Development and validation of the penn state worry questionnaire. *Behaviour Research and Therapy*, *28*(6), 487–495. https://doi.org/10.1016/0005-7967(90)90135-6

Nolen-Hoeksema, S., & Morrow, J. (1991). A prospective study of depression and posttraumatic stress symptoms after a natural disaster: The 1989 Loma Prieta earthquake. *Journal of Personality and Social Psychology*, *61*(1), 115–121. https://doi.org/10.1037/0022-3514.61.1.115

Rachman, S., Grüter-Andrew, J., & Shafran, R. (2000). Post-event processing in social anxiety. *Behaviour Research and Therapy*, *38*(6), 611–617. https://doi.org/10.1016/S0005-7967(99)00089-3

Stöber, J., & Bittencourt, J. (1998). Weekly assessment of worry: An adaptation of the Penn State Worry Questionnaire for monitoring changes during treatment. *Behaviour Research and Therapy*, *36*(6), 645–656. https://doi.org/10.1016/S0005-7967(98)00031-X

Tallis, F., Eysenck, M., & Mathews, A. (1992). A questionnaire for the measurement of nonpathological worry. *Personality and Individual Differences*, *13*(2), 161–168. https://doi.org/10.1016/0191-8869(92)90038-Q

Trapnell, P. D., & Campbell, J. D. (1999). Private self-consciousness and the five-factor model of personality: Distinguishing rumination from reflection. *Journal of Personality and Social Psychology*, *76*(2), 284–304. https://doi.org/10.1037/0022-3514.76.2.284

Treynor, W., Gonzalez, R., & Nolen-Hoeksema, S. (2003). Rumination Reconsidered: A Psychometric Analysis. *Cognitive Therapy and Research*, *27*(3), 247–259. https://doi.org/10.1023/A:1023910315561

Wells, A. (1994). A multi-dimensional measure of worry: Development and preliminary validation of the anxious thoughts inventory. *Anxiety, Stress & Coping*, *6*(4), 289–299. https://doi.org/10.1080/10615809408248803

Wells, A., & Davies, M. I. (1994). The thought control questionnaire: A measure of individual differences in the control of unwanted thoughts. *Behaviour Research and Therapy*, *32*(8), 871–878. https://doi.org/10.1016/0005-7967(94)90168-6

**SI4:** References of R toolboxes used

Balduzzi, S., Rücker, G., & Schwarzer, G. (2019). How to perform a meta-analysis with R: A practical tutorial. *Evidence Based Mental Health*, *22*(4), 153–160. https://doi.org/10.1136/ebmental-2019-300117

Harrer, M., Cuijpers, P., Furukawa, T., & Ebert, D. D. (2019). *dmetar: Companion R Package For The Guide “Doing Meta-Analysis in R.”* Retrieved from http://dmetar.protectlab.org/

Nagashima, K., Noma, H., & Furukawa, T. A. (2019). Prediction interval for random-effects meta-analysis: A confidence distribution approach. *Statistical Methods in Medical Research*, *28*(6). https://doi.org/10.1177/0962280218773520

Peterson, B. G., & Carl, P. (2020). *PerformanceAnalytics: Econometric Tools for Performance and Risk Analysis*. Retrieved from https://CRAN.R-project.org/package=PerformanceAnalytics

Pick, J. L., Nakagawa, S., & Noble, D. W. A. (2018, January 15). *Reproducible, flexible and high-throughput data extraction from primary literature: The metaDigitise* ***R*** *package*. https://doi.org/10.1101/247775

Schwarzer, G., Carpenter, J. R., & Rücker, G. (2023). *metasens: Statistical Methods for Sensitivity Analysis in Meta-Analysis*. Retrieved from https://CRAN.R-project.org/package=metasens

Viechtbauer, W., & Cheung, M. W.-L. (2010). Outlier and influence diagnostics for meta-analysis. *Research Synthesis Methods*, *1*(2), 112–125. https://doi.org/10.1002/jrsm.11

**SI5:** Final list of studies included in the meta-analysis

1. Aalderen, J. R. van, Donders, A. R. T., Giommi, F., Spinhoven, P., Barendregt, H. P., & Speckens, A. E. M. (2012). The efficacy of mindfulness-based cognitive therapy in recurrent depressed patients with and without a current depressive episode: A randomized controlled trial. Psychological Medicine, 42(5), 989–1001. https://doi.org/10.1017/S0033291711002054
2. Abdollahi, A., Hosseinian, S., Panahipour, H., & Allen, K. A. (2021). Cognitive behavioural therapy as an effective treatment for social anxiety, perfectionism, and rumination. Current Psychology, 40(9), 4698–4707. https://doi.org/10.1007/s12144-019-00411-w
3. Andersson, G., Paxling, B., Roch-Norlund, P., Östman, G., Norgren, A., Almlöv, J., … Silverberg, F. (2012). Internet-Based Psychodynamic versus Cognitive Behavioral Guided Self-Help for Generalized Anxiety Disorder: A Randomized Controlled Trial. Psychotherapy and Psychosomatics, 81(6), 344–355. https://doi.org/10.1159/000339371
4. Bell, C. J., Colhoun, H. C., Carter, F. A., & Frampton, C. M. (2012). Effectiveness of computerised cognitive behaviour therapy for anxiety disorders in secondary care. The Australian and New Zealand Journal of Psychiatry, 46(7), 630–640. https://doi.org/10.1177/0004867412437345
5. Brenes, G. A., Danhauer, S. C., Lyles, M. F., Anderson, A., & Miller, M. E. (2017). Long-Term Effects of Telephone-Delivered Psychotherapy for Late-Life GAD. The American Journal of Geriatric Psychiatry : Official Journal of the American Association for Geriatric Psychiatry, 25(11), 1249–1257. https://doi.org/10.1016/j.jagp.2017.05.013
6. Brenes, G. A., Miller, M. E., Williamson, J. D., McCall, W. V., Knudson, M., & Stanley, M. A. (2012). A Randomized Controlled Trial of Telephone-Delivered Cognitive-Behavioral Therapy for Late-Life Anxiety Disorders. The American Journal of Geriatric Psychiatry, 20(8), 707–716. https://doi.org/10.1097/JGP.0b013e31822ccd3e
7. Carl, J. R., Miller, C. B., Henry, A. L., Davis, M. L., Stott, R., Smits, J. A. J., … Espie, C. A. (2020). Efficacy of digital cognitive behavioral therapy for moderate-to-severe symptoms of generalized anxiety disorder: A randomized controlled trial. Depression and Anxiety, 37(12), 1168–1178. https://doi.org/10.1002/da.23079
8. Cheng, P., Kalmbach, D. A., Castelan, A. C., Murugan, N., & Drake, C. L. (2020). Depression prevention in digital cognitive behavioral therapy for insomnia: Is rumination a mediator? Journal of Affective Disorders, 273, 434–441. https://doi.org/10.1016/j.jad.2020.03.184
9. Cladder-Micus, M. B., Speckens, A. E. M., Vrijsen, J. N., T. Donders, A. R., Becker, E. S., & Spijker, J. (2018). Mindfulness-based cognitive therapy for patients with chronic, treatment-resistant depression: A pragmatic randomized controlled trial. Depression and Anxiety, 35(10), 914–924. https://doi.org/10.1002/da.22788
10. Corpas, J., Moriana, J. A., Venceslá, J. F., & Gálvez-Lara, M. (2022). Effectiveness of brief group transdiagnostic therapy for emotional disorders in primary care: A randomized controlled trial identifying predictors of outcome. Psychotherapy Research, 32(4), 456–469. https://doi.org/10.1080/10503307.2021.1952331
11. Costa, M. de A., Gonçalves, F. G., Tatton-Ramos, T., Fonseca, N. K. de O. da, Schwinn, J. K., Alves, S. G., … Manfro, G. G. (2020). A Three-Arm Randomized Clinical Trial Comparing the Efficacy of a Mindfulness-Based Intervention with an Active Comparison Group and Fluoxetine Treatment for Adults with Generalized Anxiety Disorder. Psychotherapy and Psychosomatics, 90(4), 269–279. https://doi.org/10.1159/000511880
12. de Almeida Sampaio, T. P., Jorge, R. C., Martins, D. S., Gandarela, L. M., Hayes-Skelton, S., Bernik, M. A., & Lotufo-Neto, F. (2020). Efficacy of an acceptance-based group behavioral therapy for generalized anxiety disorder. Depression and Anxiety, 37(12), 1179–1193. https://doi.org/10.1002/da.23021
13. Dugas, M. J., Brillon, P., Savard, P., Turcotte, J., Gaudet, A., Ladouceur, R., … Gervais, N. J. (2010). A Randomized Clinical Trial of Cognitive-Behavioral Therapy and Applied Relaxation for Adults With Generalized Anxiety Disorder. Behavior Therapy, 41(1), 46–58. https://doi.org/10.1016/j.beth.2008.12.004
14. Dugas, M. J., Ladouceur, R., Léger, E., Freeston, M. H., Langolis, F., Provencher, M. D., & Boisvert, J.-M. (2003). Group cognitive-behavioral therapy for generalized anxiety disorder: Treatment outcome and long-term follow-up. Journal of Consulting and Clinical Psychology, 71(4), 821–825. https://doi.org/10.1037/0022-006X.71.4.821
15. Dugas, M. J., Sexton, K. A., Hebert, E. A., Bouchard, S., Gouin, J.-P., & Shafran, R. (2022). Behavioral Experiments for Intolerance of Uncertainty: A Randomized Clinical Trial for Adults With Generalized Anxiety Disorder. Behavior Therapy, 53(6), 1147–1160. https://doi.org/10.1016/j.beth.2022.05.003
16. Ekkers, W., Korrelboom, K., Huijbrechts, I., Smits, N., Cuijpers, P., & Van Der Gaag, M. (2011). Competitive Memory Training for treating depression and rumination in depressed older adults: A randomized controlled trial. Behaviour Research and Therapy, 49(10), 588–596. https://doi.org/10.1016/j.brat.2011.05.010
17. Foroughi, A., Sadeghi, K., Parvizifard, A., Parsa Moghadam, A., Davarinejad, O., Farnia, V., & Azar, G. (2020). The effectiveness of mindfulness-based cognitive therapy for reducing rumination and improving mindfulness and self-compassion in patients with treatment-resistant depression. Trends in Psychiatry and Psychotherapy, 42(2), 138–146. https://doi.org/10.1590/2237-6089-2019-0016
18. Giommi, F., Castagner, V., Zaccaro, A., Gemignani, A., Serretti, A., Mandelli, L., … Chiesa, A. (2021). Mindfulness-Based Cognitive Therapy vs. Psycho-education for Patients with Anxiety Disorders Who Did Not Achieve Remission Following Adequate Pharmacological Treatment. Mindfulness, 12(8), 2059–2075. https://doi.org/10.1007/s12671-021-01664-y
19. Goldin, P. R., Morrison, A., Jazaieri, H., Brozovich, F., Heimberg, R., & Gross, J. J. (2016). Group CBT versus MBSR for Social Anxiety Disorder: A Randomized Controlled Trial. Journal of Consulting and Clinical Psychology, 84(5), 427–437. https://doi.org/10.1037/ccp0000092
20. Green, S. M., Donegan, E., McCabe, R. E., Streiner, D. L., Agako, A., & Frey, B. N. (2020). Cognitive behavioral therapy for perinatal anxiety: A randomized controlled trial. Australian & New Zealand Journal of Psychiatry, 54(4), 423–432. https://doi.org/10.1177/0004867419898528
21. Hanssen, I., Huijbers, M., Regeer, E., Bennekom, M. L. van, Stevens, A., Dijk, P. van, … Speckens, A. E. (2023). Mindfulness-based cognitive therapy v. treatment as usual in people with bipolar disorder: A multicentre, randomised controlled trial. Psychological Medicine, 53(14), 6678–6690. https://doi.org/10.1017/S0033291723000090
22. Hoyer, J., Beesdo, K., Gloster, A. T., Runge, J., Höfler, M., & Becker, E. S. (2009). Worry Exposure versus Applied Relaxation in the Treatment of Generalized Anxiety Disorder. Psychotherapy and Psychosomatics, 78(2), 106–115. https://doi.org/10.1159/000201936
23. Hyett, M. P., Bank, S. R., Lipp, O. V., Erceg-Hurn, D. M., Alvares, G. A., Maclaine, E., … McEvoy, P. M. (2018). Attenuated Psychophysiological Reactivity following Single-Session Group Imagery Rescripting versus Verbal Restructuring in Social Anxiety Disorder: Results from a Randomized Controlled Trial. Psychotherapy and Psychosomatics, 87(6), 340–349. https://doi.org/10.1159/000493897
24. Jacoby, R. J., Brown, M. L., Wieman, S. T., Rosenfield, D., Hoeppner, S. S., Bui, E., … Simon, N. M. (2023). Effect of cognitive behavioural therapy and yoga for generalised anxiety disorder on sleep quality in a randomised controlled trial: The role of worry, mindfulness, and perceived stress as mediators. Journal of Sleep Research, jsr.13992. https://doi.org/10.1111/jsr.13992
25. Kalmbach, D. A., Cheng, P., Arnedt, J. T., Anderson, J. R., Roth, T., Fellman-Couture, C., … Drake, C. L. (2019). Treating Insomnia Improves Depression, Maladaptive Thinking, and Hyperarousal in Postmenopausal Women: Comparing Cognitive-Behavioral Therapy for Insomnia (CBTI), Sleep Restriction Therapy, and Sleep Hygiene Education. Sleep Medicine, 55, 124–134. https://doi.org/10.1016/j.sleep.2018.11.019
26. Kocovski, N. L., Fleming, J. E., Hawley, L. L., Huta, V., & Antony, M. M. (2013). Mindfulness and acceptance-based group therapy versus traditional cognitive behavioral group therapy for social anxiety disorder: A randomized controlled trial. Behaviour Research and Therapy, 51(12), 889–898. https://doi.org/10.1016/j.brat.2013.10.007
27. Koszycki, D., Raab, K., Aldosary, F., & Bradwejn, J. (2010). A multifaith spiritually based intervention for generalized anxiety disorder: A pilot randomized trial. Journal of Clinical Psychology, 66(4), 430–441. https://doi.org/10.1002/jclp.20663
28. Ladouceur, R., Dugas, M. J., Freeston, M. H., Léger, E., Gagnon, F., & Thibodeau, N. (2000). Efficacy of a cognitive-behavioral treatment for generalized anxiety disorder: Evaluation in a controlled clinical trial. Journal of Consulting and Clinical Psychology, 68(6), 957–964. https://doi.org/10.1037//0022-006X.68.6.957
29. Leterme, A. C., Behal, H., Demarty, A. L., Barasino, O., Rougegrez, L., Labreuche, J., … Servant, D. (2020). A blended cognitive behavioral intervention for patients with adjustment disorder with anxiety: A randomized controlled trial. Internet Interventions, 21, 100329. https://doi.org/10.1016/j.invent.2020.100329
30. Lubbers, J., Geurts, D., Hanssen, I., Huijbers, M., Spijker, J., Speckens, A., & Cladder-Micus, M. (2022). The effect of mindfulness-based cognitive therapy on rumination and a task-based measure of intrusive thoughts in patients with bipolar disorder. International Journal of Bipolar Disorders, 10, 22. https://doi.org/10.1186/s40345-022-00269-1
31. Mennin, D. S., Fresco, D. M., O’Toole, M. S., & Heimberg, R. G. (2018). A randomized controlled trial of emotion regulation therapy for generalized anxiety disorder with and without co-occurring depression. Journal of Consulting and Clinical Psychology, 86(3), 268–281. https://doi.org/10.1037/ccp0000289
32. Monnart, A., Vanderhasselt, M.-A., Schroder, E., Campanella, S., Fontaine, P., & Kornreich, C. (2019). Treatment of Resistant Depression: A Pilot Study Assessing the Efficacy of a tDCS-Mindfulness Program Compared With a tDCS-Relaxation Program. Frontiers in Psychiatry, 10. https://doi.org/10.3389/fpsyt.2019.00730
33. Nasiri, F., Mashhadi, A., Bigdeli, I., Chamanabad, A. G., & Ellard, K. K. (2020). Augmenting the unified protocol for transdiagnostic treatment of emotional disorders with transcranial direct current stimulation in individuals with generalized anxiety disorder and comorbid depression: A randomized controlled trial. Journal of Affective Disorders, 262, 405–413. https://doi.org/10.1016/j.jad.2019.11.064
34. Newby, J. M., Williams, A. D., & Andrews, G. (2014). Reductions in negative repetitive thinking and metacognitive beliefs during transdiagnostic internet cognitive behavioural therapy (iCBT) for mixed anxiety and depression. Behaviour Research and Therapy, 59, 52–60. https://doi.org/10.1016/j.brat.2014.05.009
35. Nordahl, H. M., Borkovec, T. D., Hagen, R., Kennair, L. E. O., Hjemdal, O., Solem, S., … Wells, A. (2018). Metacognitive therapy versus cognitive–behavioural therapy in adults with generalised anxiety disorder. BJPsych Open, 4(5), 393–400. https://doi.org/10.1192/bjo.2018.54
36. Omvik, S., Sivertsen, B., Pallesen, S., Bjorvatn, B., Havik, O. E., & Nordhus, I. H. (2008). Daytime functioning in older patients suffering from chronic insomnia: Treatment outcome in a randomized controlled trial comparing CBT with Zopiclone. Behaviour Research and Therapy, 46(5), 623–641. https://doi.org/10.1016/j.brat.2008.02.013
37. Paxling, B., Almlöv, J., Dahlin, M., Carlbring, P., Breitholtz, E., Eriksson, T., & Andersson, G. (2011). Guided Internet-Delivered Cognitive Behavior Therapy for Generalized Anxiety Disorder: A Randomized Controlled Trial. Cognitive Behaviour Therapy, 40(3), 159–173. https://doi.org/10.1080/16506073.2011.576699
38. Robinson, E., Titov, N., Andrews, G., McIntyre, K., Schwencke, G., & Solley, K. (2010). Internet Treatment for Generalized Anxiety Disorder: A Randomized Controlled Trial Comparing Clinician vs. Technician Assistance. PLoS ONE, 5(6), e10942. https://doi.org/10.1371/journal.pone.0010942
39. Roemer, L., Orsillo, S. M., & Salters-Pedneault, K. (2008). Efficacy of an acceptance-based behavior therapy for generalized anxiety disorder: Evaluation in a randomized controlled trial. Journal of Consulting and Clinical Psychology, 76(6), 1083–1089. https://doi.org/10.1037/a0012720
40. Ruiz, F. J., Peña-Vargas, A., Ramírez, E. S., Suárez-Falcón, J. C., García-Martín, M. B., García-Beltrán, D. M., … Sánchez, P. D. (2020). Efficacy of a two-session repetitive negative thinking-focused acceptance and commitment therapy (ACT) protocol for depression and generalized anxiety disorder: A randomized waitlist control trial. Psychotherapy (Chicago, Ill.), 57(3), 444–456. https://doi.org/10.1037/pst0000273
41. Schoenberg, P. L. A., & Speckens, A. E. M. (2014). Modulation of induced frontocentral theta (Fm-θ) event-related (de-)synchronisation dynamics following mindfulness-based cognitive therapy in Major Depressive Disorder. Cognitive Neurodynamics, 8(5), 373–388. https://doi.org/10.1007/s11571-014-9294-0
42. Stanley, M. A., Wilson, N. L., Amspoker, A. B., Kraus-Schuman, C., Wagener, P. D., Calleo, J. S., … Kunik, M. E. (2014). Lay providers can deliver effective cognitive behavior therapy for older adults with generalized anxiety disorder: A randomized trial. Depression and Anxiety, 31(5), 391–401. https://doi.org/10.1002/da.22239
43. Stanley, M. A., Wilson, N. L., Novy, D. M., Rhoades, H. M., Wagener, P. D., Greisinger, A. J., … Kunik, M. E. (2009). Cognitive Behavior Therapy for Generalized Anxiety Disorder Among Older Adults in Primary Care: A Randomized Clinical Trial. JAMA, 301(14), 1460–1467. https://doi.org/10.1001/jama.2009.458
44. Thomas, P. J., Leow, A., Klumpp, H., Phan, K. L., & Ajilore, O. (2023). Default Mode Network Hypoalignment of Function to Structure Correlates With Depression and Rumination. Biological Psychiatry: Cognitive Neuroscience and Neuroimaging, S2451902223001854. https://doi.org/10.1016/j.bpsc.2023.06.008
45. Titov, N., Andrews, G., Johnston, L., Robinson, E., & Spence, J. (2010). Transdiagnostic Internet treatment for anxiety disorders: A randomized controlled trial. Behaviour Research and Therapy, 48(9), 890–899. https://doi.org/10.1016/j.brat.2010.05.014
46. Titov, N., Andrews, G., Robinson, E., Schwencke, G., Johnston, L., Solley, K., & Choi, I. (2009). Clinician-Assisted Internet-Based Treatment is Effective for Generalized Anxiety Disorder: Randomized Controlled Trial. Australian & New Zealand Journal of Psychiatry, 43(10), 905–912. https://doi.org/10.1080/00048670903179269
47. van der Heiden, C., Muris, P., & van der Molen, H. T. (2012). Randomized controlled trial on the effectiveness of metacognitive therapy and intolerance-of-uncertainty therapy for generalized anxiety disorder. Behaviour Research and Therapy, 50(2), 100–109. https://doi.org/10.1016/j.brat.2011.12.005
48. van der Zweerde, T., van Straten, A., Effting, M., Kyle, S. D., & Lancee, J. (2019). Does online insomnia treatment reduce depressive symptoms? A randomized controlled trial in individuals with both insomnia and depressive symptoms. Psychological Medicine, 49(3), 501–509. https://doi.org/10.1017/S0033291718001149
49. Vøllestad, J., Sivertsen, B., & Nielsen, G. H. (2011). Mindfulness-based stress reduction for patients with anxiety disorders: Evaluation in a randomized controlled trial. Behaviour Research and Therapy, 49(4), 281–288. https://doi.org/10.1016/j.brat.2011.01.007
50. Wells, A., & Colbear, J. S. (2012). Treating Posttraumatic Stress Disorder With Metacognitive Therapy: A Preliminary Controlled Trial. Journal of Clinical Psychology, 68(4), 373–381. https://doi.org/10.1002/jclp.20871
51. Wetherell, J. L., Gatz, M., & Craske, M. G. (2003). Treatment of generalized anxiety disorder in older adults. Journal of Consulting and Clinical Psychology, 71(1), 31–40. https://doi.org/10.1037/0022-006X.71.1.31
52. Winnebeck, E., Fissler, M., Gärtner, M., Chadwick, P., & Barnhofer, T. (2017). Brief training in mindfulness meditation reduces symptoms in patients with a chronic or recurrent lifetime history of depression: A randomized controlled study. Behaviour Research and Therapy, 99, 124–130. https://doi.org/10.1016/j.brat.2017.10.005
53. Wong, S. Y. S., Yip, B. H. K., Mak, W. W. S., Mercer, S., Cheung, E. Y. L., Ling, C. Y. M., … Ma, H. S. W. (2016). Mindfulness-based cognitive therapy v. group psychoeducation for people with generalised anxiety disorder: Randomised controlled trial. The British Journal of Psychiatry, 209(1), 68–75. https://doi.org/10.1192/bjp.bp.115.166124
54. Zemestani, M., Beheshti, N., Rezaei, F., Van Der Heiden, C., & Kendall, P. C. (2021). Cognitive Behavior Therapy Targeting Intolerance of Uncertainty Versus Selective Serotonin Reuptake Inhibitor for Generalized Anxiety Disorder: A Randomized Clinical Trial. Behaviour Change, 38(4), 250–262. https://doi.org/10.1017/bec.2021.16
55. Zinbarg, R. E., Eun Lee, J., & Lira Yoon, K. (2007). Dyadic predictors of outcome in a cognitive-behavioral program for patients with generalized anxiety disorder in committed relationships: A “spoonful of sugar” and a dose of non-hostile criticism may help. Behaviour Research and Therapy, 45(4), 699–713. https://doi.org/10.1016/j.brat.2006.06.005

**SI6:** Selected Characteristics of Included Studies Investigating the Effects of Cognitive-behavioral Interventions on Repetitive Negative Thinking

|  | Study Label | Primary Diagnoses | %  Female | Mean Age | *N* Post Treat | *N* Post Control | Outcome | Treatment | Speci-ficity | Setting | Ses-sions | Control Group | Control type | Qua-lity | Risk of Bias |
| --- | --- | --- | --- | --- | --- | --- | --- | --- | --- | --- | --- | --- | --- | --- | --- |
| 1 | Aalderen et al., 2012 | MDD | 65.22 | 47.50 | 34 | 35 | rumination | MBCT | gen | gr, i.-p. | 8 | TAU | passive | ++o++ | medium |
| 2 | Abdollahi et al., 2021 | SAD | 57.69 | 35.82 | 22 | 21 | rumination | CBT | gen | gr, i.-p. | 8 | WLC | passive | +-?++ | high |
| 3 | Andersson et al., 2012 | GAD | 75.93 | 42.00 | 23 | 26 | worry | AR | gen | ind, i.-b. | 8 | WLC | passive | ++o++ | medium |
| 4 | Bell et al., 2012 | SAD, PD+/-AG, GAD | 67.82 | 35.31 | 30 | 38 | worry | CCBT | gen | ind, i.-b. | 5.33 | AC | active | +++++ | low |
| 5 | Brenes et al., 2017 | GAD | 81.56 | 66.80 | 70 | 71 | worry | CBT | gen | ind, phonecall | 10 | SPT | active | ++o-- | medium |
| 6 | Brenes et al., 2012 | GAD, PD, ADNOS | 83.30 | 69.15 | 30 | 30 | worry | CBT | gen | ind, phonecall |  | PE | active | ??o++ | high |
| 7 | Carl et al., 2020 | GAD | 68.27 | 30.90 | 128 | 128 | worry | CBT | spec | ind, i.-b. | 4 | WLC | passive | +++++ | low |
| 8 | Cheng et al., 2020 | Insomnia | 78.88 | 45.05 | 358 | 300 | RNT | CBT | gen | ind, i.-b. | 4.1 | PE | active | ++o-- | medium |
| 9 | Cladder-Micus et al., 2018 | trMDD | 62.26 | 47.11 | 39 | 51 | rumination | MBCT | gen | gr, i.-p. | 8 | TAU (Pharma, PT) | active | ++o-- | medium |
| 10 | Corpas et al., 2022 | GAD, MDD, PD+/-AG, SD | 68.57 | 39.57 | 53 | 52 | worry | UP | gen | gr, i.-p. | 8 | Pharma + MC | active | +++-- | medium |
| 11.1 | Costa et al., 2020 | GAD | 78.98 | 35.90 | 76 | 79 | worry | MBI | gen | gr, i.-p. | 8 | Pharma | active | +++-- | medium |
| 11.2 |  |  |  |  |  | 68 |  |  |  |  |  | PE + SPT | active |  |  |
| 12.1 | Dugas et al., 2010 | GAD | 66.15 | 38.50 | 23 | 20 | worry | CBT-IU | spec | ind, i.-p. | 12 | AC | passive | ++o++ | medium |
| 12.2 |  |  |  |  | 22 |  |  | AR | gen | ind, i.-p. | 12 |  |  |  |  |
| 13 | Dugas et al., 2003 | GAD | 71.15 | 41.20 | 25 | 27 | worry | CBT-IU | spec | gr, i.-p. | 14 | AC | passive | ???++ | high |
| 14 | Dugas et al., 2022 | GAD | 85.00 | 34.60 | 23 | 27 | worry | CBT-IU | spec | ind, i.-p. | 12 | AC | passive | ++?++ | medium |
| 15 | Ekkers et al., 2011 | MDD | 77.17 | 72.66 | 53 | 37 | rumination | COMET | spec | gr, i.-p. | 7 | TAU (Pharma, PT) | active | +++++ | low |
| 16 | Foroughi et al., 2020 | trMDD | 72.22 |  | 10 | 12 | rumination | MBCT | gen | gr, i.-p. | 8 | Pharma | active | ----- | high |
| 17 | Giommi et al., 2021 | GAD, PD+/-AG | 54.66 | 44.93 | 23 | 21 | worry | MBCT | gen | gr, i.-p. | 8 | PE | active | ++?-+ | medium |
| 18.1 | Goldin et al., 2016 | SAD | 55.56 | 34.10 | 30 | 34 | rumination | CBT | gen | gr, i.-p. | 12 | WLC | passive | +??++ | high |
| 18.2 |  |  |  |  | 27 |  |  | MBSR | gen | gr, i.-p. | 12 |  | passive |  |  |
| 19 | Green et al., 2020 | GAD, SAD, PD+/-AG, AD | 100.00 | 31.91 | 44 | 42 | worry | CBT | gen | gr, i.-p. | 6 | WLC | passive | +++-- | medium |
| 20 | Hanssen et al., 2023 | BP-I, BP-II | 60.42 | 46.60 | 72 | 72 | rumination | MBCT | gen | gr, i.-p. | 8 | TAU | active | +++++ | low |
| 21.1 | Hoyer et al., 2009 | GAD | 71.00 | 45.40 | 27 | 29 | worry | Worry Exposure | spec | ind, i.-p. | 15 | WLC | passive | +?o++ | high |
| 21.2 |  |  |  |  | 28 |  |  | AR | gen | ind, i.-p. | 15 |  |  |  |  |
| 22.1 | Hyett et al., 2018 | SAD | 67.24 | 35.22 | 14 | 18 | RNT | CBT-imagery rescripting | gen | gr, i.-p. | 1 | WLC | passive | +++++ | low |
| 22.2 |  |  |  |  | 15 |  |  | CBT-verbal restructuring | gen | gr, i.-p. | 1 |  |  |  |  |
| 23.1 | Jacoby et al., 2023 | GAD | 70.48 | 33.83 | 67 | 59 | worry | CBT | gen | gr, i.-p. | 12 | Kundalini yoga | active | ???-- | high |
| 23.2 |  |  |  |  |  | 29 |  |  |  |  |  | Stress education | active |  |  |
| 24 | Kalmbach et al., 2019 | Insomnia | 100.00 | 56.23 | 41 | 41 | worry | CBT | gen | ind, i.-p. | 6 | PE | active | +++-- | medium |
| 25.1 | Kocovski et al., 2013 | SAD | 57.14 | 34.10 | 53 | 31 | rumination | CBT | gen | gr, i.-p. | 12 | WLC | passive | +?+++ | high |
| 25.2 |  |  |  |  | 53 |  |  | MBCT | gen | gr, i.-p. | 12 |  |  |  |  |
| 26 | Koszycki et al., 2010 | GAD | 59.09 | 43.45 | 11 | 11 | worry | Mastery of Your Anxiety and Worry | spec | ind, i.-p. | 12 | Spiritually-based Intervention | active | ???++ | high |
| 27 | Ladouceur et al., 2000 | GAD | 76.92 | 39.70 | 14 | 12 | worry | CBT-IU | spec | ind, i.-p. | 15.8 | WLC | passive | ???++ | high |
| 28.1 | Leterme et al., 2020 | ADA | 64.56 | 38.21 | 39 | 40 | worry | CBT | gen | ind, i.-p. | 5 | WLC | passive | +++-- | medium |
| 28.2 |  |  |  |  | 40 |  |  | CBT | gen | ind, mixed (i.-b.:i.-p. support) | 5 |  |  |  |  |
| 29 | Lubbers et al., 2022 | BP-I, BP-II | 63.47 | 48.38 | 14 | 15 | rumination | MBCT | gen | gr, i.-p. | 8 | TAU | active | +++-- | medium |
| 30 | Mennin et al., 2018 | GAD | 75.00 | 39.00 | 28 | 25 | worry | Emotion Regulation Therapy | gen | ind, i.-p. | 20 | AC | passive | ++o++ | medium |
| 31 | Monnart et al., 2019 | trMDD | 64.52 | 50.16 | 15 | 16 | rumination | MBCT + tDCS | gen | gr, i.-p. | 8 | tDCS + relaxation | active | ??-++ | high |
| 32 | Nasiri et al., 2020 | GAD, MDD | 73.30 | 21.03 | 15 | 15 | worry | UP | gen | ind, i.-p. | 12 | WLC | passive | +++++ | low |
| 33 | Newby et al., 2014 | GAD, MDD | 77.81 | 44.30 | 41 | 53 | RNT | iCBT | spec | ind, i.-b. | 6 | WLC | passive | ++?-- | medium |
| 34.1 | Nordahl et al., 2018 | GAD | 75.40 | 37.32 | 32 | 21 | worry | MCT | gen | ind, i.-p. | 12 | WLC | passive | +++++ | low |
| 34.2 |  |  |  |  | 28 |  |  | CBT | gen | ind, i.-p. | 12 |  | passive |  |  |
| 35 | Omvik et al., 2008 | Insomnia | 48.00 | 60.84 | 21 | 18 | worry | CBT | gen | ind, i.-p. | 6 | Pharma | active | +??+- | high |
| 36 | Paxling et al., 2011 | GAD | 79.78 | 39.29 | 44 | 45 | worry | AR + Worry Exposure | spec | ind, i.-b. | 8 | WLC | passive | ++-++ | medium |
| 37.1 | Robinson et al., 2010 | GAD | 66.31 | 47.62 | 50 | 48 | worry | Worry Program - technician assisted | spec | ind, i.-b. | 6 | WLC | passive | ++--- | medium |
| 37.2 |  |  |  |  | 47 |  |  | Worry Program - clinician assisted | spec | ind, i.-b. | 6 |  |  |  |  |
| 38 | Roemer et al., 2008 | GAD | 70.97 | 32.81 | 15 | 16 | worry | ABBT | gen | ind, i.-p. | 16 | WLC | passive | -++++ | high |
| 39 | Ruiz et al., 2020 | MDD, GAD | 70.85 | 28.5 | 18 | 24 | RNT | ACT | spec | ind, i.-p. | 2 | WLC | passive | ++-++ | medium |
| 40 | Schoenberg et al., 2014 | MDD | 62.73 | 49.47 | 26 | 25 | rumination | MBCT | gen | gr, i.-p. | 8 | WLC | passive | --?-- | high |
| 41.1 | Stanley et al., 2014 | GAD | 57.79 | 67.37 | 52 | 68 | worry | CBT | gen | ind, mixed(i.-p.:phonecall) | 7.31 | TAU | passive | +++++ | low |
| 41.2 |  |  |  |  | 60 |  |  | CBT- lay provider | gen | ind, mixed(i.-p.:phonecall) | 7.31 |  |  |  |  |
| 42 | Stanley et al., 2009 | GAD | 78.38 | 66.93 | 65 | 50 | worry | CBT | gen | ind, i.-p. | 7.4 | AC | active | +++++ | low |
| 43 | Thomas et al., 2023 | MDD, AD | 67.03 | 28.50 | 26 | 20 | rumination | CBT | gen | ind, i.-p. | 12 | Pharma | active | ++o-- | medium |
| 44 | Titov et al., 2010 | GAD, PD+/-AG, SAD | 67.97 | 39.53 | 40 | 38 | worry | Anxiety Program | gen | ind, i.-b. | 6 | WLC | passive | +--++ | high |
| 45 | Titov et al., 2009 | GAD | 76.00 | 44.00 | 24 | 21 | worry | Worry Program | spec | ind, i.-b. | 6 | WLC | passive | +??++ | high |
| 46 | Vøllestad et al., 2011 | PD+/-AG, SAD, GAD | 67.14 | 42.42 | 39 | 37 | worry | MBSR | gen | gr, i.-p. | 8 | WLC | passive | +--++ | high |
| 47 | Wells & Colbear, 2012 | PTSD | 55.00 | 37.35 | 10 | 10 | worry | MCT | gen | ind, i.-b. | 6.4 | WLC | passive | ++-++ | medium |
| 48.1 | Wetherell et al., 2003 | GAD | 80.00 | 67.10 | 18 | 18 | worry | CBT | gen | gr, i.-p. | 12 | discussion group | active | +??++ | high |
| 48.2 |  |  |  |  |  | 21 |  |  |  |  |  | WLC | passive |  |  |
| 49 | Winnebeck et al., 2017 | MDD | 60.03 | 41.52 | 36 | 32 | rumination | MBI | gen | ind, i.-p. | 3 | PE and resting | active | +++-+ | medium |
| 50.1 | Wong et al., 2016 | GAD | 78.70 | 50.59 | 61 | 61 | worry | MBCT | gen | gr, i.-p. | 8 | PE | active | +++-+ | medium |
| 50.2 |  |  |  |  |  | 48 |  |  |  |  |  | usual care | passive |  |  |
| 51 | Zemestani et al., 2021 | GAD | 100.00 | 25.14 | 15 | 15 | worry | CBT-IU | spec | ind, i.-p. | 12 | Pharma | active | +++-+ | medium |
| 52 | Zinbarg et al., 2007 | GAD | 66.67 | 41.94 | 8 | 10 | worry | Mastery of Your Anxiety and Worry | spec | ind, i.-p. | 12 | WLC | passive | +?o-+ | high |
| 53 | de Almeida Sampaio et al., 2020 | GAD | 73.91 | 36.55 | 21 | 23 | worry | ABBT | gen | gr, i.-p. | 10 | supportive group therapy | active | +??-- | high |
| 54.1 | van der Heiden et al., 2012 | GAD | 75.41 | 35.44 | 54 | 20 | worry | MCT | gen | ind, i.-p. | 14 | WLC | passive | +--++ | high |
| 54.2 |  |  |  |  | 52 |  |  | IUT | spec | ind, i.-p. | 14 |  | passive |  |  |
| 55 | van der Zweerde et al., 2019 | Insomnia | 81.75 | 45.47 | 52 | 52 | RNT | CBT | gen | ind, videocall | 5 | sleep diary | active | ++-++ | medium |

*Note.* Leftmost column: Unique study, decimal place indicates a second treatment or control arm **Primary diagnoses:** AD = Anxiety Disorder, ADNOS = Anxiety Disorder Not Otherwise Specified, ADA = Adjustment Disorder with Anxiety, AG = Agoraphobia, BP-I = Bipolar I Disorder, BP-II = Bipolar II Disorder, GAD = Generalized Anxiety Disorder, MDD = Major Depressive Disorder, PD = Panic Disorder, PTSD = Post-Traumatic Stress Disorder, SAD = Social Anxiety Disorder, SD = Somatization Disorder, trMDD = Treatment-Resistant Major Depressive Disorder**. Treatment**: ABBT = Acceptance-Based Behavior Therapy, ACT = Acceptance and Commitment Therapy, AR = Applied Relaxation, CBT = Cognitive Behavioral Therapy, IU = Intolerance of Uncertainty, IUT = Intolerance of Uncertainty Therapy, CCBT = Computerized Cognitive Behavioral Therapy, COMET = Competitive Memory Training, iCBT = Internet-Based Cognitive Behavioral Therapy, MBCT = Mindfulness-Based Cognitive Therapy, tDCS = transcranial Direct Current Stimulation, MBI = Mindfulness-Based Interventions, MBSR = Mindfulness-Based Stress Reduction, MCT = Metacognitive Therapy, UP = Unified Protocol for Transdiagnostic Treatment of Emotional Disorders. **Specificity** (treatment specificity – targeting RNT outcome explicitly): gen = general, spec = specific**. Setting**: ind = individual, gr = group, i.-b. = internet-based, i.-p. = in-person. **Control group**: AC = Attention Control, MC = Minimal Contact, Pharma = Pharmacotherapy, PE = Psychoeducation, PT = Psychotherapy, SPT = Supportive Psychotherapy, TAU = Treatment as Usual, WLC = Waitlist-Control**. Quality**: In order of appearance: (1) Proper randomized allocation, (2) allocation concealment, (3) blinding of study personnel, (4) complete outcome reporting, (5) unselective outcome reporting; “+” = yes, “-“ = no, “o” = partly, “?” = no information.

**SI7 Variable Coding as General vs. RNT-specific CBT**

| RNT-specific CBT treatments | General CBT treatments |
| --- | --- |
| ACT | ABBT |
| AR + Worry Exposure | Anxiety Program |
| CBT-IU | AR |
| COMET | (non RNT specific) CBT |
| RNT-specific iCBT | CBT - lay provider |
| IUT | CBT - imagery rescripting |
| Mastery of Your Anxiety and Worry | CBT - verbal restructuring |
| Worry Exposure | CCBT |
| Worry Program | Emotion Regulation Therapy |
| Worry Program - technician assisted | MBCT |
| Worry Program - clinician assisted | MBCT+tDCS |
|  | MBI |
|  | MBSR |
|  | MCT |
|  | UP |

ABBT = Acceptance-Based Behavior Therapy, ACT = Acceptance and Commitment Therapy, AR = Applied Relaxation, CBT = Cognitive Behavioral Therapy, IU = Intolerance of Uncertainty, IUT = Intolerance of Uncertainty Therapy, CCBT = Computerized Cognitive Behavioral Therapy, COMET = Competitive Memory Training, iCBT = Internet-Based Cognitive Behavioral Therapy, MBCT = Mindfulness-Based Cognitive Therapy, tDCS = transcranial Direct Current Stimulation, MBI = Mindfulness-Based Interventions, MBSR = Mindfulness-Based Stress Reduction, MCT = Metacognitive Therapy, UP = Unified Protocol for Transdiagnostic Treatment of Emotional Disorders.

**SI8:** Contour-enhanced funnel plot of standard error by Hedges’*g* in studies examining the effect of active CBT treatment compared with control conditions on RNT at post-test (all 69 comparisons, outliers included).

**
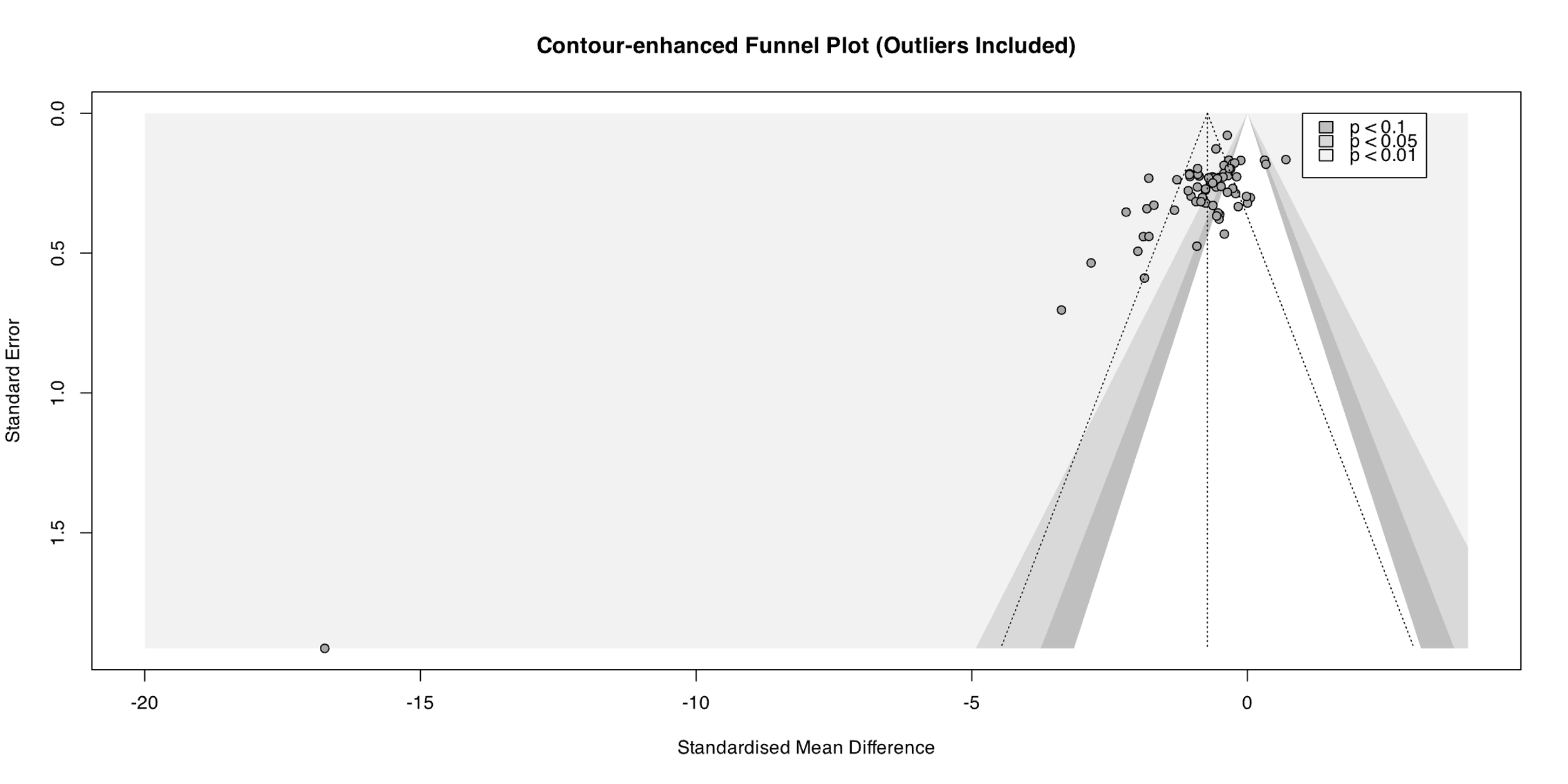
**

**SI9:** Forest plot of Hedges*’g* effect sizes of active CBT treatment compared with control conditions on RNT at post-test (all 69 comparisons, outliers included).
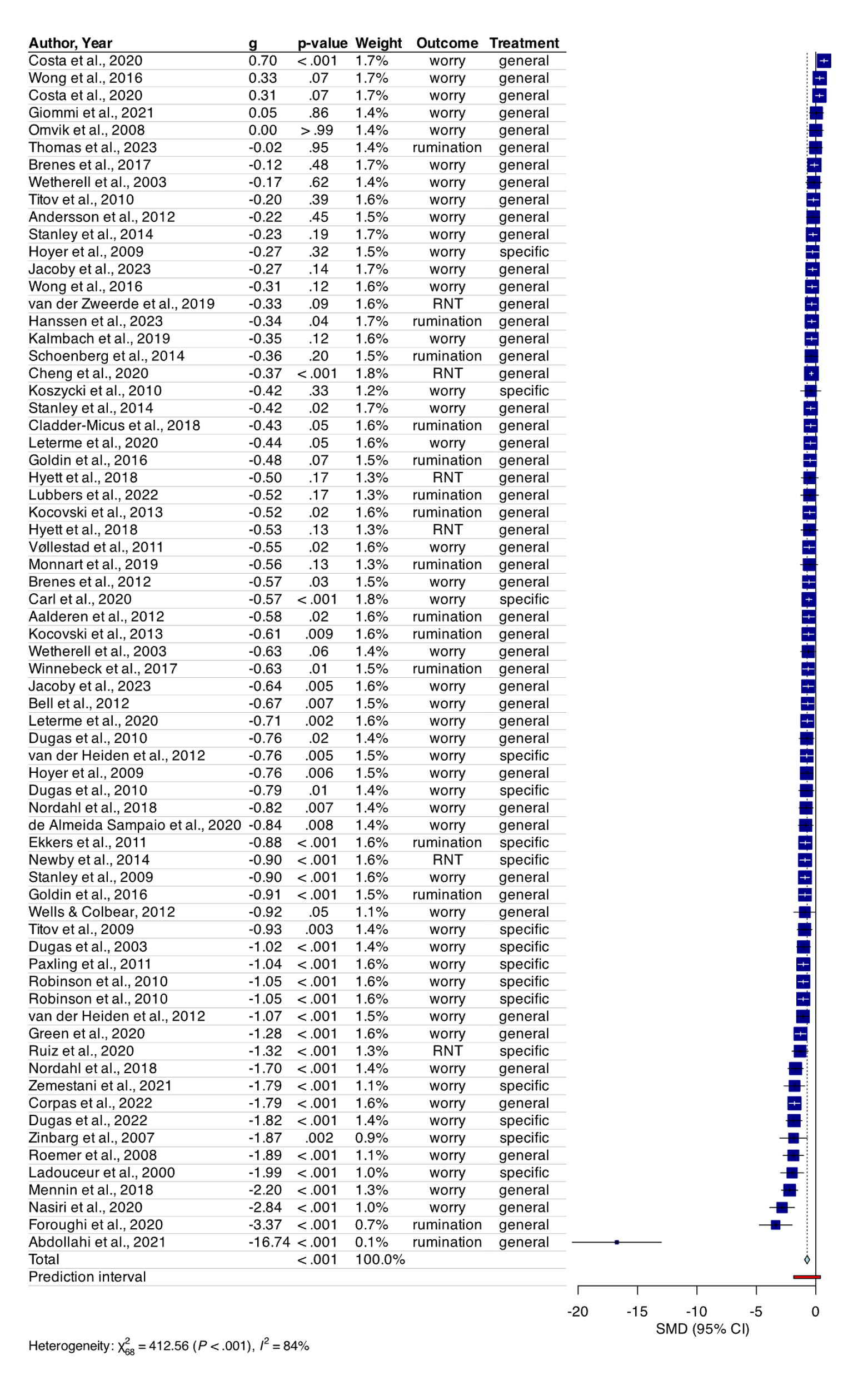


*Note.* Negative values indicate improvement of repetitive negative thinking. The position of the diamond shape indicates the average effect and its width indicates the confidence interval of the pooled result. The horizontal bar indicates the prediction interval - a range into which the effects of future studies may fall based on present evidence. Abbreviations: g = Hedge’s g. CI = Confidence Interval, LL = lower level, UL = upper level. Treatment = Treatment specificity. *X^2^* = Chi-square test of heterogeneity – higher values indicate that observed differences in can less likely be explained chance alone. *I^2^* = measure of between-study heterogeneity. SMD = Standardized Mean Difference.
